# Short-term exposure to an obesogenic diet during adolescence elicits anxiety-related behavior and neuroinflammation: modulatory effects of exogenous neuregulin-1

**DOI:** 10.1038/s41398-022-01788-2

**Published:** 2022-02-26

**Authors:** Julio David Vega-Torres, Perla Ontiveros-Angel, Esmeralda Terrones, Erwin C. Stuffle, Sara Solak, Emma Tyner, Marie Oropeza, Ike dela Peña, Andre Obenaus, Byron D. Ford, Johnny D. Figueroa

**Affiliations:** 1 Center for Health Disparities and Molecular Medicine and Department of Basic Sciences, Physiology Division, Department of Basic Sciences, Loma Linda University Health School of Medicine, Loma Linda, CA USA; 2 Department of Pharmaceutical and Administrative Sciences, Loma Linda University Health School of Pharmacy, Loma Linda, CA USA; 3 Department of Pediatrics, University of California-Irvine, Irvine, CA USA; 4 Division of Biomedical Sciences, University of California-Riverside School of Medicine, Riverside, CA USA

**Keywords:** Molecular neuroscience, Pathogenesis, Psychiatric disorders, Physiology, Predictive markers

## Abstract

Childhood obesity leads to hippocampal atrophy and altered cognition. However, the molecular mechanisms underlying these impairments are poorly understood. The neurotrophic factor neuregulin-1 (NRG1) and its cognate ErbB4 receptor play critical roles in hippocampal maturation and function. This study aimed to determine whether exogenous NRG1 administration reduces hippocampal abnormalities and neuroinflammation in rats exposed to an obesogenic Western-like diet (WD). Lewis rats were randomly divided into four groups (12 rats/group): (1) control diet+vehicle *(CDV)*; (2) CD + NRG1 *(CDN)* (daily intraperitoneal injections: 5 μg/kg/day; between postnatal day, PND 21-PND 41); (3) WD + VEH *(WDV)*; (4) WD + NRG1 *(WDN)*. Neurobehavioral assessments were performed at PND 43–49. Brains were harvested for MRI and molecular analyses at PND 49. We found that NRG1 administration reduced hippocampal volume (7%) and attenuated hippocampal-dependent cued fear conditioning in CD rats (56%). NRG1 administration reduced PSD-95 protein expression (30%) and selectively reduced hippocampal cytokine levels (IL-33, GM-CSF, CCL-2, IFN-γ) while significantly impacting microglia morphology (increased span ratio and reduced circularity). WD rats exhibited reduced right hippocampal volume (7%), altered microglia morphology (reduced density and increased lacunarity), and increased levels of cytokines implicated in neuroinflammation (IL-1α, TNF-α, IL-6). Notably, NRG1 synergized with the WD to increase hippocampal ErbB4 phosphorylation and the tumor necrosis alpha converting enzyme (TACE/ADAM17) protein levels. Although the results did not provide sufficient evidence to conclude that exogenous NRG1 administration is beneficial to alleviate obesity-related outcomes in adolescent rats, we identified a potential novel interaction between obesogenic diet exposure and TACE/ADAM17-NRG1-ErbB4 signaling during hippocampal maturation. Our results indicate that supraoptimal ErbB4 activities may contribute to the abnormal hippocampal structure and cognitive vulnerabilities observed in obese individuals.

## Introduction

Childhood obesity is a severe medical condition affecting more than 340 million children and adolescents worldwide (World Health Organization, 2016). While obesity is a complex disease, poor diet quality is a significant factor contributing to the global obesity epidemic in children and adolescents [1]. Obesity and the consumption of imbalanced obesogenic diets rich in saturated fats and simple sugars have emerged as risk factors for the development of anxiety and other stress-related disorders [2–6]. Evidence demonstrates that the relationship between obesogenic and anxiogenic phenotypes is bidirectional [7, 8]. In other words, the presence of one increases the risk of developing the other. Thus, it has become crucial to understand better the specific substrates responsible for the intertwined pathophysiology associated with both conditions.

While the neural substrates impacted by obesity are very complex, hippocampal volume deficits have emerged as a prominent anatomical endophenotype [9–12]. Studies in animals and humans indicate that the hippocampus is essential for reducing energy intake [13–15]. and stress reactivity [16, 17]. As such, alterations in this brain region may contribute to the broad range of metabolic, cognitive, and emotional disturbances associated with obesity. We reported unique hippocampal vulnerabilities to the detrimental effects of obesogenic diets [18] consistent with human data [19, 20]. However, the molecular and cellular basis underlying these vulnerabilities are poorly understood.

Obesity is associated with reduced synaptic density in humans [21] and rodents [22, 23]. Interestingly, many genes implicated with human obesity are involved in synaptic plasticity, strengthening the evidence for links between synaptic integrity and the disease [24]. Neuregulin 1 (NRG1) is a trophic factor containing an epidermal growth factor (EGF)-like domain and acting via their cognate ErbB receptor tyrosine kinases. ErbB4, the only autonomous NRG1-specific activated tyrosine kinase, plays a critical role in regulating brain development and homeostasis. NRG1-ErbB4 signaling has been heavily implicated in neuronal migration, synaptic plasticity, and cognition [25]. NRG1-ErbB4 signaling regulates hippocampal function through increasing neuronal arborization [26], increasing gamma oscillations in the CA3 region of the hippocampus [27], modulating long-term potentiation at CA1 hippocampal synapses [28], and modifying stress reactivity and behavior [29–31]. Exogenous NRG1 administration increases hippocampal neurogenesis [32, 33] and improves learning and memory while rescuing dendritic and synaptic abnormalities in a mouse model of Alzheimer’s disease (AD) [34]. Similar neurorestorative properties were reported in a rat model of AD [35]. In addition, exogenous NRG1 administration confers potent anti-inflammatory effects. We and others have demonstrated that NRG1 anti-inflammatory actions extend to models of neuroinflammation, including cerebral malaria [36], nerve agent intoxication [37], and ischemia [38]. Studies demonstrate that ErbB4 inhibition attenuates the beneficial effects of NRG1, supporting an essential role for ErbB4 in NRG1 actions [39–42].

In this study, we hypothesized that NRG1 administration would confer protection against the detrimental effects of an obesogenic diet on neuroinflammation, hippocampal maturation, and anxiety-like behaviors. We reasoned that examining the responses of the hippocampal NRG1-ErbB4 system to obesogenic diets during adolescence may provide valuable insights into the potential contribution of this pathway to obesity-induced hippocampal deficits and anxiety. This study is the first to investigate whether exogenous NRG1 possesses the therapeutic potential to prevent the early consequences of an obesogenic environment during adolescence.

## Materials and methods

### Animals

All the experiments were performed following protocol #20-171 approved by the Institutional Animal Care and Use Committee (IACUC) at the Loma Linda University Health School of Medicine. This study follows the ARRIVE guidelines for reporting animal research [43]. Female Lewis rats with 8-10 male pups (postnatal day, PND 15) were purchased from Charles River Laboratories (Portage, MI, USA). Upon arrival, female rats were housed with their pups with free access to food and water. At PND 21, adolescent male pups were weaned, housed in groups (2 per cage), and experimental manipulations commenced. Animals were kept in customary housing conditions (21 ± 2 C, relative humidity of 45%, and 12-hour light/dark cycle with lights on at 7:00 AM). The body weights were recorded once a week or daily during the week of behavioral testing. Food consumption was quantified at least twice per week. Uneaten food pellets (cage top and spillage) were weighted and recorded to the nearest 0.1 g. Subsequently, fresh food was added to the top of the cage (60 g). Food intake was expressed as an average of food weight (in kcal) per day. The food consumed was calculated at the same time of the day (0900-1100 h) for the duration of the study. The rats were never food or water restricted.

### Study Design

**Fig. 1:**
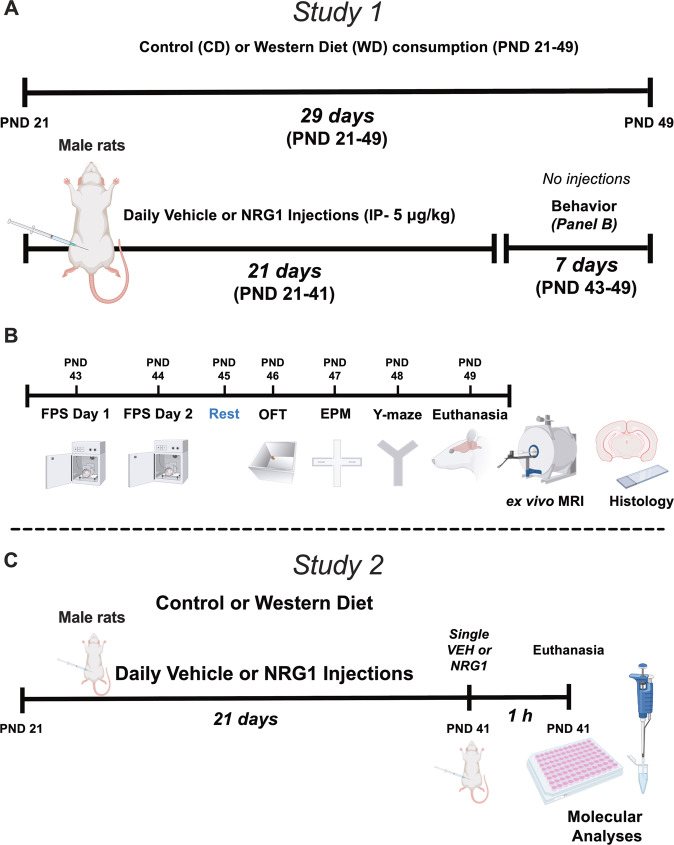
Experimental timeline and procedures. **A** Study 1 timeline and study groups. **B** Study 1 timeline of behavioral procedures, MRI, and histology. **C** Study 2 timeline of molecular analyses. Abbreviations: CD control diet, EPM elevated plus maze, FPS fear-potentiated startle, MRI magnetic resonance imaging, NRG1 neuregulin-1, OFT open field test, PND postnatal day, VEH vehicle, WD Western diet.

We used a 2 × 2 design with four groups: (1) Control diet, injected with vehicle (CD + VEH); (2) Control diet, injected with neuregulin-1 (CD + NRG1); (3) Western diet, injected with vehicle (WD + VEH); (4) Western diet, injected with neuregulin-1 (WD + NGR1). Figure 1 summarizes the timeline of experimental procedures and behavioral tests. The matched low-fat purified control diet (CD, 5-gm% fat, product *#F7463*) and Western-like high-saturated fat diet (WD, 20-gm% fat, product *#F7462*) were obtained from Bio-Serv (Frenchtown, NJ, USA). The macronutrient composition and fatty acid profiles are detailed in a previous study and summarized in Supplemental Table 1 [44]. Weight-matched adolescent rats (PND 21) were divided into four study groups: CD + VEH, CD + NRG1, WD + VEH, and WD + NRG1. NRG1 (5 micrograms/kilogram/day) or saline (vehicle) intraperitoneal injections were performed daily between 12:00-1:00 PM. The rationale for injection dosage and route was based on our previous reports describing the neuroprotective effects of this intervention [37, 45], NRG1 biodistribution, kinetics, and short half-life in plasma [46, 47]. Recombinant human NRG1-β 1/HRG1-β 1 EGF domain (NRG1) was provided by Dr. Byron D. Ford (manufactured by R&D Systems, catalog #*396-HB-050*). Study 1 (Fig. 1A,B: behavioral and anatomical assessment): All behavior testing sessions involved a 20-30-minute acclimation period to the testing facility. The rats were allowed to consume the custom diets until the completion of the study (PND 21-49). Guided by prior research [48], the initial group size was *n* = 18 for each group (divided into four cohorts). Twenty-four rats were randomly selected for MRI studies (*n* = 6 per group). Study 2 (Fig. 1C: molecular markers investigation): To minimize potential carryover effects between behavioral tests and molecular outcomes, we used a separate group of animals without behavioral manipulations for the molecular outcomes reported in this study (Western blot and flow cytometry). For this group, an additional injection of NRG1 or saline was administered 1 h before euthanizing the animals at PND 42 [46]. The initial group size for Study 2 was *n* = 5–6 for each group (CD + VEH, *n* = 5; CD + NRG1, *n* = 5; WD + VEH, *n* = 6; WD + NRG1, *n* = 6). Experimenters were blinded to the treatment conditions. Figure 1 summarizes the experimental procedures for each study.

### Study 1

#### Behavioral test battery

We use a specific test battery to investigate phenotypic behavioral responses to stressors in rats that consume obesogenic diets. Our standard battery includes acoustic startle (and its plasticity: habituation, PPI), conditioned fear, open field activity, Y maze, and elevated plus-maze. Generally, behavioral tests are run from least invasive to most invasive to minimize behavioral reactivity based on prior test history (carryover effects). However, a fundamental goal of our investigations is to understand precisely how previous experiences alter behavior. Specifically, we are interested in understanding how adolescent dietary obesity influences stress reactivity [18, 44, 48, 49]. Thus, in our battery, we have used foot shocks not only to promote fear conditioning but also as a stressor [44, 48, 49], as has been reported by others [50–52]. In our hands, this sensitive and comprehensive test battery (and testing order) is a powerful tool to unmask the impact of an obesogenic diet on fear and anxiety-like responses.

### Fear-potentiated startle (FPS)

#### FPS rationale

The startle reflex is influenced by the corticolimbic system and modulated by both attentional and emotional states. Thus, the classic form of startle plasticity or fear-potentiated startle (FPS) represents a robust and reliable tool to assess cognitive and emotional aspects of fear reactivity. Given our prior work identifying the detrimental effects of the obesogenic diet on hippocampal structure and function [18], we used a trace FPS paradigm to evaluate hippocampal-dependent cued fear learning.

#### FPS apparatus

Startle responses were assessed inside the SR-Lab acoustic chambers (San Diego Instruments, Inc., San Diego, CA, USA). Each chamber contained a Plexiglas enclosure with a calibrated piezoelectric motion sensor. The startle reflex has a nonzero baseline and is graded in magnitude. Accelerometer readings were obtained at 1 ms intervals for 200 ms after the startle-inducing acoustic stimulus and recorded using SR-LAB startle software. Each enclosure had grid floors capable of delivering foot shocks (San Diego Instruments, CA).

#### FPS Training Session

During this Pavlovian fear conditioning paradigm, a neutral stimulus (light) elicits increases in the acoustic startle response after pairings with a foot shock. Each FPS session started with a 5-min acclimation period (background noise = 60 dB). During the first session of the paradigm (fear training), the rats were trained to associate a light stimulus (conditioned stimulus, CS) with a 0.6 mA foot shock (unconditioned stimulus, US). The conditioning session involved 10 CS + US pairing presentations. During each CS + US presentation, the onset of the shock (500 ms duration) occurred 3 s after the offset of the 4 s light. This interval has been shown to recruit the hippocampus while producing similar startle responses as those observed in delay conditioning (REF). Light-shock pairings were presented in a quasi-random manner (ITI = 3-5 min).

#### FPS Testing Session

The cued fear acquisition was measured 24 h later. The testing session started with a brief 5 min acclimation period (background noise = 60 dB). During cued fear acquisition testing (fear learning testing), the rats were first presented with 15 startle-inducing tones (leaders; 5 each at 90 dB, 95 dB, and 105 dB) delivered alone at 30 s ITI. These leader trials were used to familiarize the rats with the acoustic stimuli, facilitating more accurate startle reactivity measurement during the session. Subsequently, the rats were presented with 60 test trials. For half of these test trials, a 20 ms tone was presented alone (tone alone trials; 10 trials for each tone: 90 dB, 95 dB, and 105 dB). For the other half, the tone onset (20 ms duration) occurred 3 s after the offset of the 4 s light; 10 trials for each pairing: 90 dB, 95 dB, and 105 dB. The 60 trials were divided into 10 blocks of six test trials each, including three tone alone trials and three light + tone trials. To conclude the testing session, the rats were presented with 15 startle-inducing tones (trailers; five each at 90 dB, 95 dB, and 105 dB) delivered at 30 s ITI. These trailer trials are commonly compared to the leader trials and used to investigate startle habituation during the testing session. All the trials in this session were presented in a quasi-random order (ITI = 30 s). The startle-eliciting tones had a 20 ms duration. For each animal, we calculated the mean startle magnitude in the absence (tone alone startle) and in the presence of the learned light stimulus (light + tone startle) and the percent difference between these means.

#### FPS analyses and interpretation

Enclosure movements (Foot Shock Reactivity) were recorded during the training session to investigate differences in sensory reactivity to the aversive foot shock. This measure provides critical information for interpreting FPS data (e.g., if the diet or treatment attenuates the acquisition of fear-potentiated startle, is this effect related to a potential analgesic effect during training so that the foot shocks fail to promote associative learning?).

We assessed fear acquisition with data from the testing session. This was achieved by comparing the startle amplitude from light + tone trials (conditioned + unconditioned stimulus, CS + US) relative to tone alone trials (unconditioned stimulus, US). FPS data was reported as the change (delta) between US and CS + US [FPS = (Light + Tone Startle) − (Tone Alone Startle)]. Conditioned fear responses that deviated from control animals were associated with potential hippocampal-related psychopathology. Increased potentiated startle responses (relative to controls) usually indicate heightened fear, while decreased FPS suggests deficits in fear acquisition, retention, and/or expression.

### Open field test (OFT)

The OFT was performed in an opaque acrylic open field maze (43.2 cm length × 43.2 cm width × 30.5 cm height). The rats were placed in the center of the field and recorded for 5 min using Ethovision XT (Noldus Information Technology). Behaviors were recorded at normal room lighting conditions (269 lux illumination). We used Ethovision to subdivide the arena into nine zones, which allowed for the evaluation of inner (center) and outer arena exploration. The chamber was cleaned with 70% ethanol between trials.

### Elevated plus maze (EPM)

The near-infrared (NIR)-backlit EPM apparatus (catalog #ENV-564A, MedAssociates) consisted of two opposite open arms (50.8 × 10.2 cm) and two enclosed arms (50.8 × 10.2 × 40.6 cm) that were elevated 72.4 cm above the floor. Behaviors were recorded under low light conditions (<10 lx). The rat was placed on the central platform facing an open arm and allowed to explore the maze for 5 min. The apparatus was thoroughly cleaned after each test session. Behaviors were recorded via a monochrome video camera equipped with a NIR filter. The time spent on both types of arms, the number of entries and duration in both types of arms, the latency to the first entry into any of the open arms (OA), closed arms (CA) entries, frequency in the head-dipping zone, and stretch attend postures (SAPs) were determined using Ethovision XT tracking software (Noldus Information Technology). From these data, we calculated the anxiety index according to our group and others [18, 44, 53, 54]. Anxiety index = 1 − [(OA cumulative duration/total test duration) + (OA entries / total number of entries to CA + OA)]/2.

### Y-maze spontaneous alternation

The Y-maze apparatus was constructed of black Plexiglass with three identical arms spaced at an angle of 120 degrees (arms dimensions: 55.9 cm L × 11.4 cm W × 29.2 cm H). Each rat was placed at the end of the start arm and allowed to move freely through the maze during a nine-min session. The total number of arm entries and alternations from visited to unvisited arms was recorded using a video camera, tracked using Ethovision XT (Noldus Information Technology), and scored manually by two blinded investigators. Entries occur when all four limbs are within the arm. Each arm choice was recorded, and spontaneous alternation behaviors were scored as three successive choices that include one instance of each arm (a triad). The percentage of spontaneous alternations was calculated as the total number of alternations/(total arm entries − 2) x 100. This measure is considered to reflect short-term memory in rats. The alternate arm returns and same arm returns were also scored for each animal to investigate attention within spontaneous working memory. The number of total alternations was calculated as an index of locomotor activity, and rats exhibiting 0 entries were excluded from analyses. Behaviors were recorded at normal room lighting conditions (269 lx). The maze was cleaned with 70% ethanol between trials.

### Magnetic resonance imaging

All fixed brain tissues underwent ex vivo high-resolution imaging using a 9.4 T Bruker Avance Imager (Bruker Biospin, Billerica, MA). All the data were collected with a 2.2-cm field of view, 0.55-mm slice thickness, and a 200 × 180 acquisition zero-filled to a final 256 × 256 matrix. T2-weighted RARE (rapid acquisition with relaxation enhancement; T2 RARE) were collected with the following parameters: repetition time (TR)/echo time (TE) = 6482 msec/49.3 msec, RARE factor of 4, for a final resolution of 110 x 111 x 550 microns. The total imaging time was 39 min.

The experimenters were blinded to the group designation during MRI analysis. MRI scans were eddy corrected, and the cranium was removed using MatLab. The Waxholm rat brain atlas was fit to each animal, and regional labels were applied with Advanced Normalization Tools (ANTs). Regional volumes were extracted for 72 regions (144 bilateral regions). All data were extracted and summarized in Excel. Given our interest in hippocampal atrophy, we focused on hippocampal volumes and subfields.

### Neurohistology

#### Tissue preparation and immunohistochemistry

Before euthanasia, the animals were deeply anesthetized with an intraperitoneal overdose injection of Euthasol (150 mg/kg; Virbac, Fort Worth, TX) and sacrificed via transcardiac perfusion with 10% sucrose (isotonic at 9.25% in distilled water; prewash) and 4% paraformaldehyde (PFA; fixative) using the Perfusion Two^TM^ Automated Pressure Perfusion System (Leica Biosystems, Buffalo Grove, IL). Following MRI, a subgroup of brains was randomly selected from each study group for histological analyses (*n* = 12; three rats per group). The brains were removed from the cranial vault after MRI and washed with phosphate-buffered saline (PBS) and cryoprotected with sucrose (30%) for 12–16 h at 4 °C before embedding in Tissue-Tek^®^ O.C.T.^TM^ compound (Sakura, Torrance, CA, United States). Three (3) brains per group were later sectioned with a cryostat (Cryocut 1800 Cryostat, Leica Biosystems, Vista, CA, USA) at 25 μm thickness in the coronal plane. Regions of interest (ROI) included dorsal and ventral areas of the CA1 region of the hippocampus of both hemispheres with a distance from Bregma between −4.6 and −5.6. The sectioning pattern followed a 1:5 series (one 25-μm section was collected every four sections. Three (3) consecutive brain sections encompassing the ROIs were used for immunohistochemistry. Sections were mounted in glass slides, and immunohistochemistry staining was performed with the microglia/macrophage marker, ionized calcium-binding adaptor protein molecule 1 (Iba-1). First, sections were incubated in a quenching buffer (10% methanol, 1% hydrogen peroxide in PBS) to block endogenous peroxide activity. To block the signal from endogenous avidin, biotin, and biotin-binding proteins in tissue, we used Avidin/Biotin blocking kit (Abcam, USA) followed by incubation with normal serum (5% Donkey and 5% Goat serum) with 1% Triton-X in PBS. Sections were then incubated overnight with a polyclonal rabbit anti-Iba1 primary antibody (1:750, rabbit anti-Iba1, catalog #019-19741, Wako, USA) followed by secondary biotinylated antibody incubation (1:200, goat anti-rabbit IgG, Vector Labs Inc.). Controls for nonspecific binding consisted in omitting the primary antibody (Supplemental Fig. 6C). The Vectastain Elite ABC HRP kit and DAB peroxidase substrate kit with nickel (Vector Labs Inc., Burlingame, CA, USA) were used to visualize staining according to manufacturer instructions. Following staining, slides were dehydrated in ethanol (79, 95, and 100%), rinsed in Xylene, sealed with Permount Mounting Medium (Fisher Chemical), and coverslips applied for subsequent visualization.

#### Image acquisition and segmentation processing for microglial morphological characterization

Blinded investigators carried out image acquisition and analyses. Images of tissue sections that were DAB-stained with the Iba-1 antibody were obtained using a Keyence BZ-9000 series microscope (Keyence Corporation, Osaka, Japan) in brightfield at a 40× magnification. To obtain a single high-quality full-focused composite image that showed detailed magnification of ramified processes of the cells and facilitated image segmentation, a multi-plane virtual-Z mode with 10 images (1 μm thickness) in 20 μm depth of tissue section was captured using the BZ-9000 BIOREVO sectioning algorithm. Images were later combined using public domain software *ImageJ* (https://imagej.nih.gov/ij/). Hippocampal dorsal and ventral CA1 areas were scanned to obtain at least 3 images per region of interest per hemisphere covering a field of view area of 1.2 mm. Each acquired image was saved as a TIFF file and included at least six (6) Iba-1+ cells.

Images were processed in an unbiased and systematic way to obtain a filled image. For this purpose, a series of steps were performed using ImageJ with the Fiji plugin (https://imagej.net/Fiji). First, a single high-quality image was obtained by merging all the single images and processing them under a minimum threshold feature to soften the background and enhance the contrast. Subsequently, the image was transformed to 8-bit grayscale and binarized to obtain a black and white image by applying a previously established threshold. To obtain a cell image consisting of continuous pixels, manual editing of each image was performed. A set threshold of pixel number addition (no more than 4 pixels) to join processes belonging to a selected cell was established and performed systematically. A 2-pixel set threshold was applied to separate ramification to neighboring cells when applicable. This step was carefully performed under the view of the original z-stack images to avoid bias. The final filled shape image was saved and later analyzed with *FracLac* V2.5 for *ImageJ* (Karperien, A., FracLac for ImageJ; http://rsb.info.nih.gov/ij/plugins/fraclac/FLHelp/Introduction.htm) [55] to quantify the morphological changes of microglial cells in each experimental group. Foreground pixel quantification of the filled binary images per object (cell) was done with the Box Counting (BC) feature, which counts the number of boxes containing any foreground pixels along with the successively smaller caliber grids. BC box size scale was obtained as a Power Series where the base is raised to the exponent added to it to make successive sizes. Finally, the slope for each object was the average of 12 measurements with different and random grid placements. Box Counting and Convex and Hull Area measurements were exported using Excel (Microsoft, Redmond, WA, USA) for record and tabulation. Graphpad Prism version 9.0 (Graphpad Software, La Jolla, CA, USA) was used for statistical analyses. We conducted power analyses using *G*∗*Power* [56]. For repeated measures two-way ANOVA (with diet type and treatment as factors and four measurements per group), post-hoc analyses revealed that three rats per group are sufficient to detect medium effect sizes (*d* = 0.475) with power (1 - β) set at 0.80, and *α* = 0.05. The statistical power (1-β) for the microglial morphometric parameters data was 0.16 for detecting a small size effect (*d* = 0.2), whereas the power exceeded 0.99 for the detection of large effect size (*d* = 0.8). This indicates that the histological experiments are adequately powered at or above a moderate size level (*d* = 0.4).

### Study 2

#### Western Blot

We used a separate group of animals without behavioral manipulations for Western blot experiments to minimize potential carryover effects of behavioral tests on molecular outcomes (CD + VEH, *n* = 5; CD + NRG1, *n* = 5; WD + VEH: *n* = 6; WD + NRG1, *n* = 6). For this group, an additional injection of NRG1 or saline was administered 1 h before euthanizing the animals to investigate the acute activation of NRG1-ErbB4 signaling pathways. The rats were euthanized with intraperitoneal administration of Euthasol (Virbac) and perfused transcardially with PBS. The rats were rapidly decapitated, the brains were isolated, and the left hippocampus was dissected. The tissue was immediately homogenized in 600 μL MPER Extraction Buffer (Thermo, 78501) (supplemented with protease and phosphatase inhibitors) using Bullet Blender. Homogenates were centrifuged for 10 min at 14,000 x g at 4 °C. The supernatant was removed, aliquoted, and stored at −80 °C until the day of the experiment. Protein concentration was determined using the Pierce BCA Protein Assay (Thermo, 23225). Homogenates were then mixed with 4x Protein Sample Loading Buffer (LI-COR Biosciences, 928-40004), NuPAGE Sample Reducing Agent (10x) (Thermo, NP0009), and subsequently heated at 95 °C for 5 min. Fifty micrograms (50 µg) of protein were separated on NuPAGE 4–12% Bis-Tris gels (Invitrogen). Proteins were transferred to iBlot Gel Transfer Stacks Nitrocellulose Membranes (Invitrogen) using the iBlot 2 System (Thermo). The blots were washed and blocked for 1 h in 5% dry milk or Intercept (TBS) blocking buffer (LI-COR, 927-60001) and incubated with primary antibodies overnight at 4 °C (diluted in 1x TBS with 0.1% Tween® 20). Primary antibody dilutions are described in Supplemental Table 2. Blots were subsequently washed in 1x TBS with 0.05% Tween® 20 and incubated with appropriate fluorescent secondary antibodies (LI-COR; diluted at 1:30,000 in 1x TBS with 0.1% Tween® 20) for 90 min at room temperature. Membranes were washed with 1x TBS before imaging. Images were obtained using Odyssey® Fc Imager (LI-COR).

### Bead-based cytokine detection and metabolic status measurements

To examine neuroinflammation from hippocampal homogenates, we performed a multianalyte bead-based immunoassay (LEGENDplex^TM^ Custom Panel of Rat Inflammation markers; Biolegend, San Diego, CA, USA) to provide fast and quantitative information on the WD and NRG1 treatment effects on the concentration of relevant pro and anti-inflammatory cytokines and chemokines. Rats were euthanized with an intraperitoneal administration of Euthasol (Virbac, Fort Worth, TX, USA) and perfused transcardially with ice-cold PBS (same animals used for Western blot analyses). Animals were rapidly decapitated, and the brains and plasma were isolated. For total protein extraction, right hippocampal brain tissue was homogenized in a buffer containing 20 mmol/L Tris-HCl (pH 7.5), 150 mmol/L NaCl, 1 mmol/L PMSF, 0.05% Tween-20, and a Protease Inhibitor Cocktail (cOmplete™ Mini Protease Inhibitor Cocktail, Roche Diagnostic, USA) (CD + VEH, *n* = 5; CD + NRG1, *n* = 5; WD + VEH: *n* = 6; WD + NRG1, *n* = 6). Samples were then agitated for 30 min at 4 °C and centrifuged at 12,000 rpm for 20 min at 4 °C; the supernatant was then removed. Protein concentration was quantified in triplicates using the DC™ Protein Assay (Bio-Rad Laboratories, Hercules, CA, USA) according to the manufacturer’s instructions, and absorbance was read at 655 nm using Spectramax i3x detection platform (Molecular Devices, Sunnyvale, CA, USA).

Cytokine evaluation of hippocampal and plasma IL-10, IL-1α, IL-6, IL-1β, TNF-α, IL-33, GM-CSF, CXCL-1, CCL2, IL-18, IL-12p70, IL-17a, and INF-γ were quantified in triplicates using the bead-based Assay LEGENDplex^TM^ protocol for V bottom plate, according to the manufacturer’s instructions (intra-assay coefficient of variation, CV%, was 26.7). Samples were diluted at 1:4 with Assay Buffer. Cytokine concentrations were determined using antibodies for each analyte covalently conjugated to a set of microspheres in a carbodiimide crosslinking reaction and detected by a cocktail of biotinylated antibodies. Following binding of streptavidin–phycoerythrin conjugate, the fluorescent reporter signal was measured using a MACSQuant® Analyzer 10 (Miltenyi Biotech, Bergisch Gladbach, Germany). FCS files were evaluated with the LEGENDplex™ Data Analysis Software Suite using the Qognit cloud base platform (Biolegend). A five-parameter logistic curve-fitting method was used to calculate concentrations from Median Fluorescence Intensity and normalized to the amount of protein in each sample. The results are reported as pg/mg of protein.

### Metabolic and inflammatory assessments to examine obesity status

Please refer to published work from our lab for detailed protocols on the metabolic biomarker measures used in this study [18]. Blood glucose: We report circulating glucose concentration following active-cycle ad libitum feeding as a proxy for early metabolic imbalances (glycemic responses). Post-prandial blood glucose concentration was measured in anesthetized rats by cutting the tail tip right before inducing euthanasia. Glucose concentration was measured using a glucometer (OneTouch UltraMini® manufactured by LifeScan Inc, Milpitas, CA, USA) and reported as mg per dL. Study 2 rats were used for the molecular experiments (same rats used for Western blot analyses). Plasma was collected and stored at −80 °C. Corticosterone: Circulating plasma concentration of corticosterone (CORT) was measured by ELISA (Enzo Life Sciences, ADI-900-097 Ann Arbor, MI, USA) according to the manufacturer’s instructions. Plasma samples were diluted with kit assay buffer (1:5 dilutions) and ran in triplicates. The absorbance was measured at 405 nm with 570 nm correction using the SpectraMax i3X detection platform (Molecular Devices, Sunnyvale, CA). Corticosterone concentration was determined as the percentage bound using a standard curve with a detection range between 20,000 and 31.25 pg/mL. Values are reported as picograms per milliliter. Leptin: Plasma samples were used to evaluate leptin concentration, samples were diluted with kit assay buffer (1:3 dilutions), ran in triplicates, and measurements were made using an enzyme-linked immunosorbent assay ELISA kit (Leptin Rat ELISA Kit Abcam, ab100773; Cambridge, MA, USA) according to manufacturer’s instructions. Plates were read at 450 nm using the SpectraMax i3X (Molecular Devices, Sunnyvale, CA USA). Specific concentrations for each sample were determined as mean absorbance using a standard curve of samples ranging from 0 to 8,000 pg/mL. Values were then calculated and reported as picograms of leptin per mL. Insulin: Plasma samples were assayed for insulin content using an insulin ELISA kit (Abcam, ab100578, Cambridge, MA, USA) following the manufacturer’s instructions. Samples were diluted with Assay Diluent (1:5 dilution) in triplicates, and concentration was measured against a standard curve from 0 to 300 uIU. Samples were read at 450 nm using the SpectraMax i3X (Molecular Devices, Sunnyvale, CA, USA). Insulin concentration is reported as uIU per mL. Triglycerides: Plasma samples were used to determine triglycerides concentration with the TG assay kit for quantification (Abcam, ab65336; Cambridge, MA, USA) as previously described [18]. Samples were diluted in with kit assay buffer (1:3 dilution) and ran in triplicates. Triglyceride concentration was determined using the SpectraMax i3X plate reader (Molecular Devices, Sunnyvale, CA USA). TG concentration was determined as the mean absorbance at 570 nm using a standard curve of samples ranging from 0 to 10,000 pmol. TG concentration was calculated and reported as milligrams of TG per dL. FGF-21: Plasma samples were assayed for FGF-21 concentration using an ELISA kit from R&D Systems (MF2100, R&D Systems, Minneapolis MN, USA) according to the manufacturer’s instructions. Samples were diluted with Assay Diluent (1:2 dilution) in triplicates and concentration measured against a standard curve from 0 to 2,000 pg/mL. Samples were read at 450 nm and corrected at 540 nm using the SpectraMax i3X (Molecular Devices, Sunnyvale, CA USA). FGF-21 concentration is reported as ng/mL. NRG1: Hippocampal homogenate samples were used to determine NRG1 concentration with the Human NRG1-β1 ELISA (DY377, R&D Systems, Minneapolis MN, USA). Samples were diluted in kit assay buffer (1:6 dilution) in triplicates. NRG1 concentrations were determined using the SpectraMax i3X plate reader (Molecular Devices, Sunnyvale, CA USA) as the mean absorbance at 450 nm with correction at 540 nm using a standard curve of samples ranging from 0 to 4,000 pg/mL. NRG1 concentration was calculated and reported as nanograms of NRG1 per mL.

### Statistics

We analyzed the data using GraphPad *Prism* version 9.0 (Graphpad Software, La Jolla, CA, USA). Shapiro-Wilk statistical analyses were used to determine sample distribution. The Brown–Forsythe test was used to test for the equality of group variances. Two-way analysis of variance (ANOVA) was used to examine the effect of the diet type, treatment, and interaction between factors on outcome measures. Three-way ANOVA was used to investigate the impact of the diet type, treatment, hippocampal regions, and interactions between factors on microglial morphology. Mixed-effects model analyses were used to examine the effect of the diet type, treatment, ErbB4 isoform type, and interactions on hippocampal ErbB4 mRNA levels. Multiple comparisons were made using Sidak’s (for one significant main effect; within-group) or Tukey’s test (for more than one significant main effect or significant interaction effect; between-group). The ROUT method was used to determine outliers. Principal component analyses (PCA) were performed to reduce the microglia morphological descriptors’ dimensionality by using centering/scaling methods. We considered differences significant when *p* < 0.05. The data is shown as the mean ± standard error of the mean (SEM).

### Images and figures

Illustrations were prepared using BioRender (www.Biorender.com), OmniGraffle Pro (The Omni Group; Seattle, WA), and Prism (Graphpad Software; La Jolla, CA).

## Results

### Early impact of macronutrient profile of diet on biometric parameters

Following the reports indicating that exogenous NRG1 improves metabolic outcomes implicated with obesity [47, 57–60], we tested whether exogenous NRG1 administration prevents/delays the onset of obesity symptoms. We postulated that exogenous NRG1 administration would attenuate the WD-induced hippocampal structural impairments and alterations in cued learning, spatial working memory, inflammatory signals, and synaptic integrity. Figure 1 details the experimental timeline and experimental groups (CDV, control diet + vehicle injections; CDN, control diet + NRG1 injections; WDN, Western diet + vehicle injections; WDN, Western diet + NRG1 injections). Here, we found that adolescent rats exposed to the Western-like high-fat diet (WD) for three weeks exhibited significantly increased body weight, caloric intake, and post-prandial blood glucose concentration relative to the rats that consumed the control diet (CD) (*p* < 0.05; Table 1). However, three weeks of consuming the obesogenic WD was not sufficient to significantly alter critical peripheral obesity biomarkers, including leptin, insulin, triglycerides, and corticosterone (*p* > 0.05; Table 1). Subchronic exogenous NRG1 administration had no significant effects on the biometric parameters assessed in this study (*p* > 0.05; Table 1).

**Table 1:** Detailed biometric parameters findings and statistics.

Parameter	CD		WD		*F*	*P*
	VEH	NRG1	VEH	NRG1		
Body weight (g)	146.08 ± 2.30	146.88 ± 2.37	155.37 ± 1.88	157.03 ± 2.30	Interaction: *F* (1, 66) = 0.03	*P* = 0.85
					Treatment: *F* (1, 66) = 0.30	*P* = 0.58
					Diet: *F* (1, 66) = 19.20	***P*** < **0.0001**
Caloric intake (kcal/d)	52.84 ± 0.73	53.09 ± 0.96	63.39 ± 1.59	66.09 ± 1.04	Interaction: *F* (1, 20) = 1.18	*P* = 0.30
					Treatment: *F* (1, 20) = 1.71	*P* = 0.21
					Diet: *F* (1, 20) = 109.20	***P*** < **0.0001**
PBG (mg/dl)	128.08 ± 2.77	132.08 ± 3.08	144.92 ± 4.32	139.75 ± 4.04	Interaction: *F* (1, 44) = 1.61	*P* = 0.21
					Treatment: *F* (1, 44) = 0.026	*P* = 0.87
					Diet: *F* (1, 44) = 11.50	***P*** = **0.0015**
Insulin (uIU/mL)	38.92 ± 1.85	41.23 ± 4.82	34.23 ± 3.17	37.47 ± 3.87	Interaction: *F* (1, 18) = 0.017	*P* = 0.90
					Treatment: *F* (1, 18) = 0.45	*P* = 0.45
					Diet: *F* (1, 18) = 0.26	*P* = 0.26
Corticosterone (ng/mL)	18.01 ± 1.66	19.46 ± 3.79	23.40 ± 3.65	20.94 ± 3.06	Interaction: *F* (1, 18) = 0.36	*P* = 0.55
					Treatment: *F* (1, 18) = 0.02	*P* = 0.87
					Diet: *F* (1, 18) = 1.13	*P* = 0.30
Leptin (pg/mL)	597.57 ± 57.96	515.13 ± 22.62	640.33 ± 32.80	532.77 ± 57.07	Interaction: *F* (1, 18) = 0.075	*P* = 0.79
					Treatment: *F* (1, 18) = 4.27	*P* = *0.05*
					Diet: *F* (1, 18) = 0.43	*P* = 0.52
Triglycerides (mg/dL)	118.35 ± 0.57	119.84 ± 0.93	119.48 ± 1.33	114.80 ± 3.13	Interaction: *F* (1, 18) = 1.94	*P* = 0.18
					Treatment: *F* (1, 18) = 0.32	*P* = 0.32
					Diet: *F* (1, 18) = 0.43	*P* = 0.43
FGF-21 (ng/mL)	0.72 + /- 0.07	0.59 + /- 0.07	0.91 + /- 0.13	1.12 + /- 0.11	Interaction: *F* (1, 18) = 2.75	*P* = 0.11
					Treatment: *F* (1, 18) = 0.70	*P* = 0.70
					Diet: *F* (1, 18) = 11.57	***P*** = **0.003**

The adolescent rats that consumed the WD exhibited an increase in body weight, food consumption, circulating FGF-21 levels, and post-prandial blood glucose levels relative to animals consuming the control diet at postnatal day 41. Three weeks of consuming the WD was not sufficient to alter plasma insulin, leptin, corticosterone, and triglyceride levels in adolescent rats. Exogenous NRG-1 administration did not have a significant effect on any of the biometric parameters measured. Bold denotes significant effects following two-way ANOVA. Sample numbers: Body weight: CD + VEH, *n* = 17; CD + NRG1, *n* = 17; WD + VEH, *n* = 18; WD + NRG1, *n* = 18. Caloric intake: CD + VEH, *n* = 6; CD + NRG1, *n* = 6; WD + VEH, *n* = 6; WD + NRG1, *n* = 6. PBG: CD + VEH, *n* = 12; CD + NRG1, *n* = 12; WD + VEH, *n* = 12; WD + NRG1, *n* = 12. Insulin, CORT, Leptin, TG, and FGF21: CD + VEH, *n* = 5; CD + NRG1, *n* = 5; WD + VEH, *n* = 6; WD + NRG1, *n* = 6.

### Exogenous NRG1 decreases trace cued fear acquisition in the adolescent rats that consume the CD but not in the rats that consumed the obesogenic WD

Recent work from our lab has focused on identifying early (mal)adaptive responses to obesogenic environments [18, 44, 48, 49, 61]. Having demonstrated that exposure to an obesogenic diet during adolescence alters the neural and behavioral substrates implicated with emotional regulation, we asked whether these impairments can be attenuated by administering neuregulin-1 (NRG1), a neurotrophic factor with potent neuroprotective and anti-inflammatory properties [38, 41, 45, 62].

**Fig. 2:**
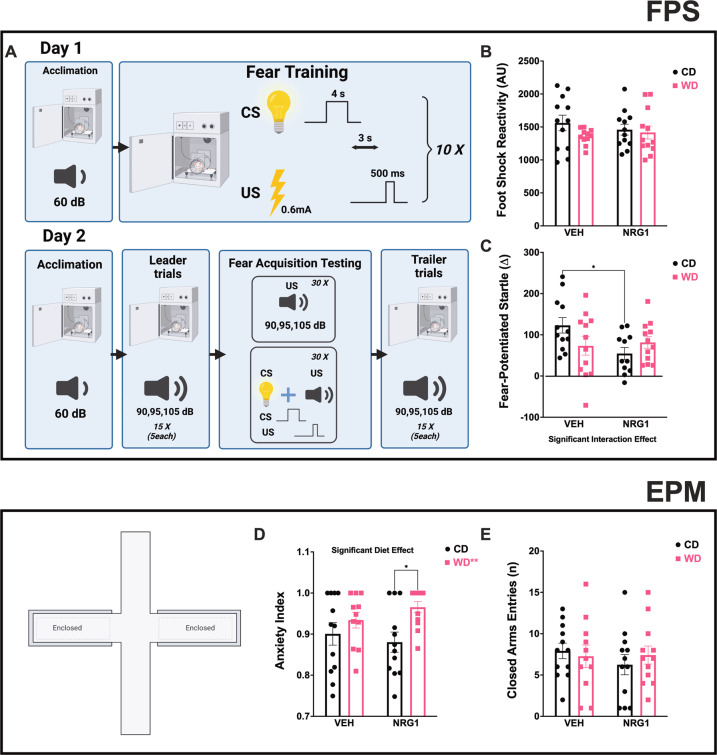
Behavioral phenotypes associated with early access to an obesogenic diet and prolonged NRG1 administration. **A** Schematic illustration of trace FPS protocol. **B** Startle reactivity to the foot shocks was not affected by treatment [*F*_(1, 43)_ = 0.05, *p* = 0.82] or diet [*F*_(1, 43)_ = 1.82, *p* = 0.19]. **C** Fear potentiated-startle (FPS) responses were determined from trace conditioning protocol and showed a decrease in FPS in CDN rats relative to CDV (interaction: [*F*_(1, 43)_ = 4.57, *p* = 0.038], post hoc, *p* = 0.021). **D** Elevated-plus maze (EPM) anxiety index was affected by the diet [*F*_(1, 43)_ = 7.27, *p* = 0.009]. WDN rats demonstrated higher anxiety indices in the EPM when compared to CDN (*p* = 0.017). **E** Number of closed arms entries in the EPM was not affected by treatment [*F*_(1, 43)_ = 0.434, *p* = 0.514] or diet [*F*_(1, 43)_ = 0.051, *p* = 0.822]. For all behaviors: sample size = 12 rats/group (before outlier testing).

Trace conditioning is thought to reflect hippocampal-dependent declarative memory [63–65]. Hippocampal lesions impair acquisition and expression of trace conditioning, while having little effect on the acquisition of amygdalar-dependent delay conditioning [66, 67] Based on our findings reporting structural impairments in hippocampus of rats that consumed an obesogenic diet during adolescence [18], we decided to investigate the effect of the WD and exogenous NRG1 on a trace conditioning paradigm (Fig. 2A). We found no significant interaction [*F*_(1, 43)_ = 0.79, *p* = 0.38], treatment [*F*_(1, 43)_ = 0.05, *p* = 0.82], or diet [*F*_(1, 43)_ = 1.82, *p* = 0.19] effects on foot shock reactivity (Fig. 2B). Interestingly, daily NRG1 administration (5 μg/kg/day for 21 days between postnatal day, PND21-PND41) led to a significant reduction in fear-potentiated startle (FPS) responses at 24 h following trace cued fear conditioning. Analyses showed a significant interaction [*F*_(1, 43)_ = 4.57, *p* = 0.038] effect in FPS responses, with no significant treatment [*F*_(1, 43)_ = 2.81, *p* = 0.10] or diet [*F*_(1, 43)_ = 0.40, *p* = 0.53] (Fig. 2C). Post hoc testing revealed that this effect was unique to the animals that consumed the CD (significant differences between the CDV and CDN rats, *p* = 0.021). When analyzing each separate tone intensity, we found a stimulus effect (tone alone vs. light + tone) for 90 dB [*F*_(1, 40)_ = 64.82, *p* < 0.0001], 95 dB [*F*_(1, 44)_ = 27.71, *p* < 0.0001], and 105 dB [*F*_(1, 43)_ = 37.61, *p* < 0.0001]. Interestingly, only CDV rats exhibited significant differences between the stimulus type (Supplemental Fig. 2). This phenotype is consistent with previous reports from our lab proposing that obesity and the consumption of obesogenic diets saturates fear and emotional circuits [48].

### WD consumption during adolescence increases indices of anxiety in the EPM

Numerous studies demonstrate that dietary obesity is associated with anxiety-related phenotypes [18, 68, 69]. To assess stress and anxiety-related responses, we employed a test battery, including the FPS, open-field test (OFT), elevated plus maze (EPM), and Y-maze. Echoing prior findings, analyses revealed a significant diet [*F*_(1, 43)_ = 7.27, *p* = 0.009], and no significant interaction [*F*_(1, 43)_ = 1.38, *p* = 0.25] or treatment [*F*_(1, 43)_ = 0.066, *p* = 0.80] effects on the EPM anxiety index (Fig. 2D). WDN rats demonstrated higher anxiety indices in the EPM when compared to CDN (*p* = 0.017). We used the number of closed arm entries as a proxy for locomotion in the EPM. We found no significant interaction [*F*_(1, 43)_ = 0.613, *p* = 0.438], treatment [*F*_(1, 43)_ = 0.434, *p* = 0.514], or diet [*F*_(1, 43)_ = 0.051, *p* = 0.822] effects in the number of closed arm entries (Fig. 2E). Additional OFT, EPM, and Y-maze behavioral outcomes are included in Supplemental Fig. 3.

### Exogenous subchronic NRG1 reduces the hippocampal volume in adolescent rats that consume the CD but not in the rats that consume the obesogenic WD

**Fig. 3:**
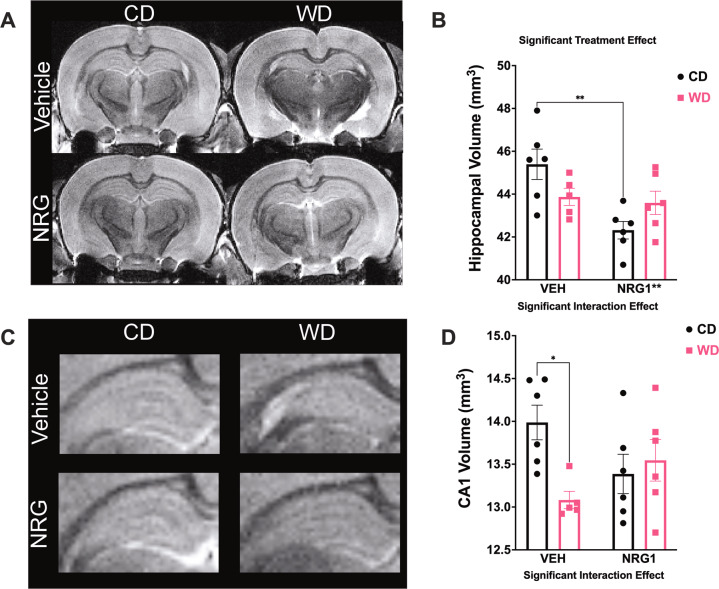
Obesogenic diet and prolonged NRG1 administration interact to influence hippocampal volumes. **A**, **B** Total hippocampal volume (both left and right hippocampi combined) were affected by the treatment [*F*_(1, 19)_ = 9.48, *p* = 0.006]. Subchronic NRG1 administration during adolescence resulted in significant total hippocampal volume reduction (CDV vs. CDN, *p* = .003). **C**, **D** Hippocampal subfield segmentation showed a significant interaction [*F*_(1, 19)_ = 6.44, *p* = 0.020] effects on CA1 region. WDV rats had a significantly smaller CA1 volume than CDV rats (*p* = 0.015). Sample size = 6 rats/group.

Exogenous NRG1 confers robust anti-inflammatory [36, 37], neuroprotective [41, 45, 62], and neurogenic effects [32, 33]. In this respect, we asked whether NRG1 administration prevents hippocampal atrophy in rats that consume obesogenic diets. We examined hippocampal volumetric measurements using magnetic resonance imaging (MRI). We found a significant interaction [*F*_(1, 19)_ = 6.61, *p* = 0.019] and treatment [*F*_(1, 19)_ = 9.48, *p* = 0.006] effects on total hippocampal volume (both left and right hippocampi combined), with no diet [*F*_(1, 19)_ = 0.052, *p* = 0.82] effects (Fig. 3A, B). The rats that received prolonged NRG1 administration during early adolescence exhibited smaller hippocampi (CDV vs. CDN, *p* = 0.003), an effect that was dependent on the diet type. The volumetric differences were not related to changes in total brain volume. Analyses revealed no significant interaction [*F*_(1, 19)_ = 0.14, *p* = 0.71], treatment [*F*_(1, 19)_ = 0.18, *p* = 0.67], or diet [*F*_(1, 19)_ = 0.25, *p* = 0.62] effects on total brain volume (Supplemental Fig. 4).

Hippocampal subfield segmentation revealed unique vulnerabilities to the experimental manipulations in the CA1 region. Analyses revealed a significant interaction [*F*_(1, 19)_ = 6.44, *p* = 0.020] and no significant treatment [*F*_(1, 19)_ = 0.11, *p* = 0.75] or diet [*F*_(1, 19)_ = 3.16, *p* = 0.092] effects (Fig. 3C, D), indicating a crossover interaction between the main factors. WDV rats had a significantly smaller CA1 volume than CDV rats (*p* = 0.035). Table 2 details hippocampal volumetric data and statistics. The left and right rodent hippocampi exhibit striking anatomical and functional lateralization. Similarly, it has been well-established that the human brain exhibits neuroanatomical differences between the left and right sides [70]. The human hippocampus shows normative structural asymmetries [71], reflected in differences in function and behavior between both hemispheres. Notably, studies demonstrate enhanced hippocampal asymmetry in cognitive decline, Alzheimer’s disease, and dementia [72]. In general, increased lateralization has been associated with cognitive deficits [73]. Thus, changes in the typical pattern of hippocampal asymmetry have been implicated in several pathological states and proposed to serve as neuroanatomical markers of risk. We dissected the effects of the WD and hippocampal hemispheric lateralization (Supplemental Fig. 5C). We found significant hippocampal hemisphere lateralization [*F*_(1, 19)_ = 6.08, *p* = 0.023] and diet type [*F*_(1, 19)_ = 4.71, *p* = 0.043] effects, with no significant interaction [*F*_(1, 19)_ = 0.053, *p* = 0.82] effects on hippocampal volumetrics. WD rats exhibited a reduced right hippocampal volume relative to the left in CD rats (post hoc *p* = 0.019). We found a significantly increased lateralization in the hippocampus of rats that consumed the WD, suggesting a novel potential neuropathological signature of early obesity (Supplemental Fig. 5C). Interestingly, the WD rats that received exogenous NRG1 did not exhibit hippocampal laterality (Supplemental Fig. 5D).

**Table 2:** **A** Detailed right hippocampal subfield volumetrics and statistics. **B** Detailed left hippocampal subfield volumetrics and statistics.

	CD VEH		CD NRG		WD VEH		WD NRG	
	Mean	SEM	Mean	SEM	Mean	SEM	Mean	SEM
Total	44.42	0.64	**41.39**	0.61	43.02	0.50	42.81	0.76
CA1	14.07	0.18	**13.11**	0.24	13.78	0.27	13.60	0.30
CA2	0.56	0.04	0.49	0.05	0.51	0.04	0.61	0.03
CA3	11.83	0.27	**11.06**	0.19	11.46	0.21	11.30	0.24
DG	12.56	0.22	**11.58**	0.28	12.10	0.17	12.17	0.28
SUB	5.40	0.13	5.16	0.11	5.17	0.11	5.14	0.06

Source of Variation	*F* (DFn, DFd)	*P* value
Region	*F* (1.647, 32.93) = 13763	<**0.0001**
Diet	*F* (1, 20) = 0.0001678	0.9898
Drug	*F* (1, 20) = 6.457	**0.0194**
Region x Diet	*F* (5, 100) = 0.1115	0.9896
Region x Drug	*F* (5, 100) = 5.106	**0.0003**
Diet x Drug	*F* (1, 20) = 4.937	**0.0380**
Region x Diet x Drug	*F* (5, 100) = 3.751	**0.0037**
Subject SS, 32.29		

	CD VEH		CD NRG		WD VEH		WD NRG	
	Mean	SEM	Mean	SEM	Mean	SEM	Mean	SEM
Total	46.37	1.09	**43.24**	0.92	44.63	0.27	44.37	0.72
CA1	13.90	0.30	**12.99**	0.21	13.48	0.28	13.49	0.43
CA2	0.88	0.06	0.76	0.04	0.84	*0.02*	0.83	0.07
CA3	11.95	0.48	11.11	0.22	11.71	0.15	11.50	0.25
DG	13.44	0.19	**12.38**	0.36	13.12	0.28	12.70	0.09
SUB	6.20	0.18	5.99	0.18	6.16	0.20	5.85	0.14

Source of variation	*F* (DFn, DFd)	*P* value
Region	*F* (1.452, 29.03) = 8810	<**0.0001**
Diet	*F* (1, 20) = 0.001380	0.9707
Drug	*F* (1, 20) = 5.423	**0.0305**
Region x Diet	*F* (5, 100) = 0.02894	0.9996
Region x Drug	*F* (5, 100) = 4.558	**0.0009**
Diet x Drug	*F* (1, 20) = 1.597	0.2209
Region x Diet x Drug	*F* (5, 100) = 1.527	0.1882
Subject SS, 60.71		

Bold denotes significant effects following three-way ANOVA and post hoc comparisons between CD + VEH and CD + NRG1. Subject refers to the differences among subjects (variation source expressed as Sum of Squares, SS). Sample numbers: CD + VEH, *n* = 6; CD + NRG1, *n* = 6; WD + VEH, *n* = 6; WD + NRG1, *n* = 6. Bold denotes significant effects following three-way ANOVA and post hoc comparisons between CD + VEH and CD + NRG1. Subject refers to the differences among subjects (variation source expressed as Sum of Squares, SS). Sample numbers: CD + VEH, *n* = 6; CD + NRG1, *n* = 6; WD + VEH, *n* = 6; WD + NRG1, *n* = 6.

### Quantitative morphometric analysis shows a diverse morphologic microglia response to an obesogenic diet and exogenous NRG1 treatment in the CA1 region of the hippocampus

**Fig. 4:**
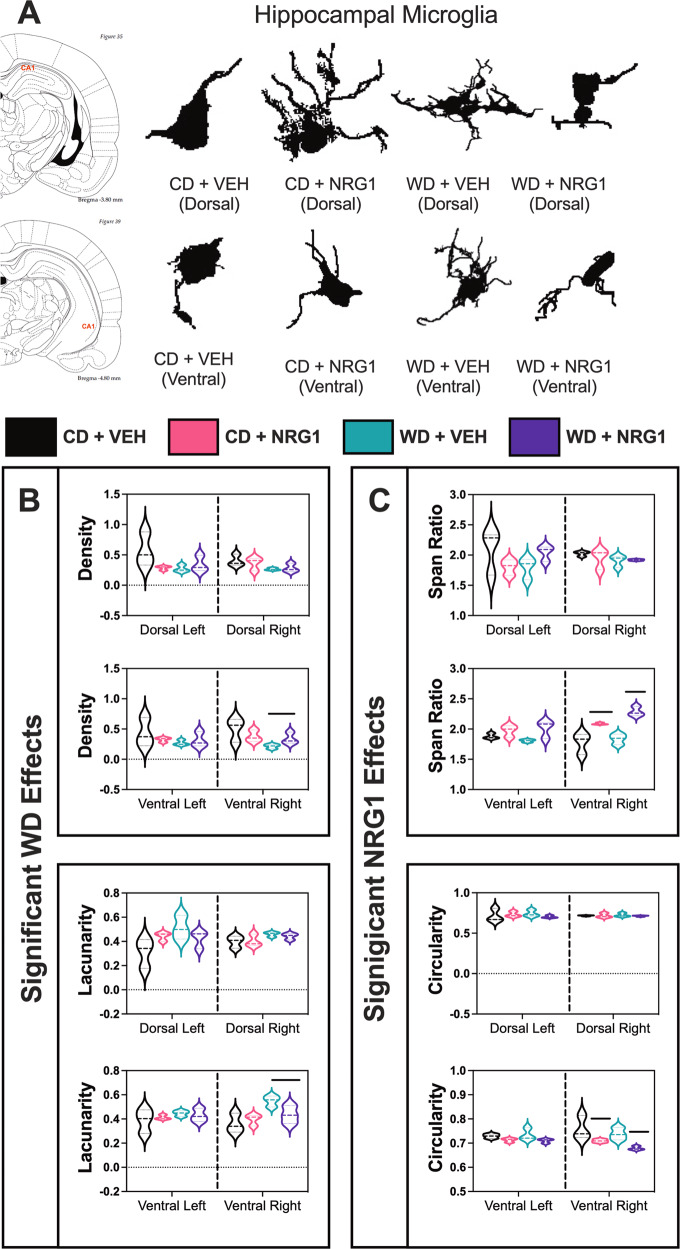
Obesogenic diet and exogenous NRG1 injections alter microglial shape in the hippocampus. Full-focused composite 40x images were acquired from twelve (12) rats. Hippocampal dorsal and ventral CA1 areas were scanned to obtain at least three (3) images per region of interest per hemisphere covering a field of view area of 1.2 mm. Each acquired image was saved as a TIFF file and included at least six (6) Iba-1^+^ cells. **A** Representative microglia morphologies from hippocampal dorsal and ventral CA1 regions in each study group. **B** Diet effects on hippocampal microglia morphology: evaluation of microglial *density* as a measure of cell size based on area demonstrated that the WD significantly decreased microglial density in the right CA1 subfield (diet: [*F*_(1,8)_ = 6.82, *p* < 0.031]). *Lacunarity* was significantly increased in the right CA1 subfield of rats that consumed an obesogenic WD (diet: [*F*_(1,8)_ = 12.09, *p* < .008]). **C** Exogenous NRG1 effects on hippocampal microglia morphology: the span ratio was significantly increased in the right CA1 region of rats receiving the exogenous NRG1 (treatment: [*F*_(1,8)_ = 15.43, *p* < 0.004]). NRG1 administration reduced *circularity* in the right CA1 region (treatment: [*F*_(1,8)_ = 9.67, *p* < 0.014]). Sample size = approximately 500 microglia per group; 3 rats/group.

Microglia are the innate immune cells of the brain. Notably, microglia have been shown to play a significant role in axonal remodeling and synaptic pruning [74], and hippocampal tissue volume [75]. Microglia are highly responsive to obesogenic diets [22, 23] and NRG1 [38, 76]. Given that microglial function is closely related to its morphology [77], we measured an array of 18 key morphometric parameters using fractal analysis (Supplemental Table 4). We focused on the CA1 hippocampal subfield based on its role on hippocampal-dependent behavior, abundant ErbB4 expression, and the observed volumetric changes and vulnerabilities of this region to several environmental insults. We performed morphological analyses in 1,981 Iba-1-positive cells (approximately 500 microglia/group). We applied Principal Component (PC) Analysis on the morphometric parameters to trace the possible differences in microglia driving hippocampal structure and behavior changes. PCs with eigenvalues greater than 1 were used (*Kaiser* rule). For all groups, we had three PCs with eigenvalues greater than 1. These PCs explained more than 80% of the accumulated variance between cells (PC1: ~60%; PC2: ~20%; PC3: ~5%; Supplemental Fig. 7). The analyses allowed us to extract presumably independent (non-overlapping) factors reflecting different parameters constituting microglial shape and function in our rat model. We identified several morphometric features with variable contribution values higher than .06 (1/18; 18 total number of features). These morphometric parameters were subjected to PCA. PCA revealed distinctive morphological profiles between groups; PC1 contributed to explaining diet effects, and PC2 helped understand the differences between NRG1-treated groups (PC1 = 58, PC2 = 35%; Supplemental Fig. 8). Figure 4A shows representative microglia morphologies from hippocampal dorsal and ventral CA1 regions in each study group. Evaluation of microglial density (solidity) as a measure of cell size based on area demonstrated that the WD significantly decreased microglial density in the right CA1 subfield (Fig. 4B) (ROI: [*F*_(1,8)_ = 1.06, *p* = 0.33]; *diet*: [*F*_(1,8)_ = 6.82, *p* = 0.031]; treatment: [*F*_(1,8)_ = 0.01, *p* = 0.93]; ROI x diet: [*F*_(1,8)_ = 0.82, *p* = 0.39]; ROI x treatment: [*F*_(1,8)_ = 0.04, *p* = 0.84]; diet x drug: [*F*_(1,8)_ = 2.17, *p* = 0.18]; ROI x diet x drug: [*F*_(1,8)_ = 2.71, *p* = 0.14]). This finding suggests a less compact/more ramified microglia phenotype. Microglial lacunarity is a measure of heterogeneity that reflects sensitive changes in particular features such as soma size relative to process length [78]. We found that lacunarity was significantly increased in the right CA1 subfield of rats that consumed an obesogenic WD (Fig. 4B) (ROI: [*F*_(1,8)_ = 0.21, *p* = 0.65]; diet: [*F*_(1,8)_ = 12.09, *p* = 0.008]; treatment: [*F*_(1,8)_ = 0.93, *p* = 0.36]; ROI x diet: [*F*_(1,8)_ = 2.77, *p* = 0.13]; ROI x treatment: [*F*_(1,8)_ = 0.74, *p* = 0.42]; diet x drug: [*F*_(1,8)_ = 3.30, *p* = 0.11]; ROI x diet x drug: [*F*_(1,8)_ = 2.76, *p* = 0.14]), suggesting higher cellular complexity.

The span ratio is a measure that describes the cell shape (or form factor) and is based on the ratio of length and height of the Convex Hull area occupied by the cell [77]. The higher the span ratio, the less difference in area between soma and processes (elongated morphotype). The span ratio was significantly increased in the right CA1 region of rats receiving the exogenous NRG1 (Fig. 4C) (*ROI*: [*F*_(1,8)_ = 1.43, *p* = 0.27]; diet: [*F*_(1,8)_ = 0.41, *p* = 0.54]; *treatment:* [*F*_(1,8)_ = 15.43, *p* < 0.004]; ROI x diet: [*F*_(1,8)_ = 6.88, *p* = 0.03]; ROI x treatment: [*F*_(1,8)_ = 29.31, *p* = 0.001]; diet x drug: [*F*_(1,8)_ = 1.63, *p* = 0.24]; ROI x diet x drug: [*F*_(1,8)_ = 0.11, *p* = 0.75]). Circularity is a measure of shape (soma thickness). High circularity values are associated with round sphere shapes, suggesting a more ameboid-like morphology associated with greater phagocytic activity [77]. NRG1 administration reduced circularity (induced irregular shape) in the right CA1 region (Fig. 4C) (ROI: [*F*_(1,8)_ = 0.14, *p* = 0.72]; diet: [*F*_(1,8)_ = 1.62, *p* = 0.24]; treatment: [*F*_(1,8)_ = 9.67, *p* < 0.015]; ROI x diet: [*F*_(1,8)_ = 1.81, *p* = 0.22]; ROI x treatment: [*F*_(1,8)_ = 6.00, *p* = 0.04]; diet x drug: [*F*_(1,8)_ = 0.33, *p* = 0.58]; ROI x diet x drug: [*F*_(1,8)_ = 0.007, *p* = 0.94]). Analyses revealed significant interaction effects for Max/Min Raddi (ROI: [*F*_(1,8)_ = 1.23, *p* = 0.30]; *diet:* [*F*_(1,8)_ = 0.049, *p* = 0.83]; treatment: [*F*_(1,8)_ = 3.17, *p* < 0.011]; ROI x diet: [*F*_(1,8)_ = 5.52, *p* = 0.047]; ROI x treatment: [*F*_(1,8)_ = 13.04, *p* = 0.007]; diet x drug: [*F*_(1,8)_ = 3.56, *p* = 0.10]; ROI x diet x drug: [*F*_(1,8)_ = 0.040, *p* = 0.85]) and CV from all Raddi from Circle’s Center (ROI: [*F*_(1,8)_ = 2.16, *p* = 0.18]; diet: [*F*_(1,8)_ = 0.03, *p* = 0.87]; treatment: [*F*_(1,8)_ = 0.90, *p* < 0.37]; ROI x diet: [*F*_(1,8)_ = 3.57, *p* = 0.10]; ROI x treatment: [*F*_(1,8)_ = 5.95, *p* = 0.041]; diet x drug: [*F*_(1,8)_ = 1.53, *p* = 0.25]; ROI x diet x drug: [*F*_(1,8)_ = 1.16, *p* = 0.31]) in the right CA1 hippocampal subfield. Complete details regarding microglial morphology descriptors definitions can be found in Supplemental Table 4. Detailed microglial parameter values were reported as mean ± SEM for each group (Supplemental Table 5).

### WD consumption increases cytokine concentrations while exogenous NRG1 administration decreases selective cytokine concentrations in the rat hippocampus

**Fig. 5:**
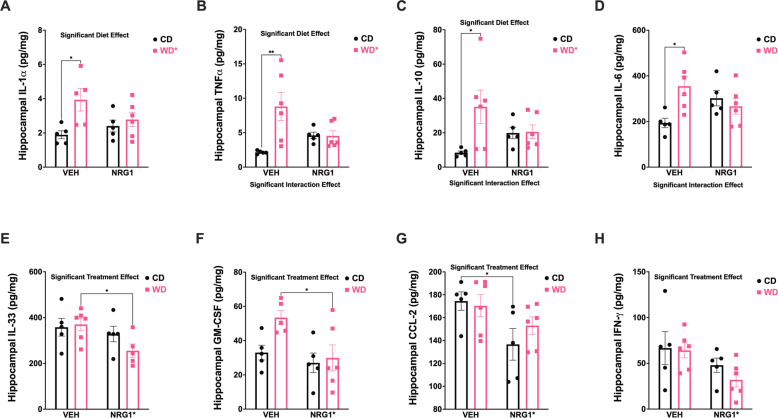
Obesogenic diet consumption and prolonged NRG1 injections alter selective inflammatory mediators in the rat hippocampus. Bead-based flow cytometry immunoassay was used to evaluate cytokine levels. **A**–**D** The WD led to a significant increase in cytokines in the hippocampus: IL1-α (*p* = 0.010), TNF-α (*p* = 0.006), IL-10 (*p* = 0.027), and IL-6 (*p* = 0.019). **E**–**H** NRG1 administration significantly reduced IL-33 (*p* = 0.041), GM-CSF (*p* = 0.021), CCL-2 (*p* = 0.037), and IFN-γ (*p* = 0.031). Supplemental Table 3 includes the detailed statistics of the cytokines listed in Fig. 5. Sample size = 5–6 rats/group. Sample numbers: CD + VEH, *n* = 6; CD + NRG1, *n* = 6; WD + VEH, *n* = 6; WD + NRG1, *n* = 6.

Obesity is associated with a robust neuroinflammatory state via increased inflammatory mediators. Our data revealing diet-induced changes in microglial morphology prompted us to examine hippocampal cytokine concentrations. Using a bead-based flow cytometry immunoassay, we found that the WD exerted a precise cytokine regulation (Fig. 5). The rats that consumed the WD exhibited a significant increase in pro-inflammatory cytokines in the hippocampus: IL1-α (*p* = 0.010), TNF-α (*p* = .006), and IL-6 (*p* = 0.019). Interestingly, the anti-inflammatory cytokine, IL-10, was also significantly increased in WD rats (*p* = 0.027). Opposite to the main dietary effects in increasing hippocampal cytokine concentrations, exogenous NRG1 administration significantly reduced IL-33 (*p* = 0.041), GM-CSF (*p* = 0.021), CCL-2 (*p* = 0.037), and IFN-γ (*p* = 0.031), confirming the potent immunomodulatory effects of NRG1 [37, 38].

Notably, WD and NRG1 influenced the concentrations of several peripheral cytokines and inflammatory mediators (Supplemental Fig. 9). Exogenous NRG1 decreased IL-10 plasma concentration (diet: [*F*_(1, 16)_ = 2.38, *p* = 0.14], treatment [*F*_(1, 16)_ = 15.71, *p* = 0.0011]; interaction: [*F*_(1, 16)_ = 0.17, *p* = 0.69]) (Supplemental Fig. 9A). We found that exogenous NRG1 administration reduced IL-17A concentration in plasma (diet: [*F*_(1, 15)_ = 0.57, *p* = 0.46], treatment [*F*_(1, 15)_ = 7.18, *p* = 0.017]; interaction: [*F*_(1, 15)_ = 2.02, *p* = 0.18]) (Supplemental Figure 9B). Interestingly, we found that while the obesogenic WD increased the concentration of CXCL1, exogenous NRG1 elicited a marked reduction in the levels of this chemokine (diet: [*F*_(1, 16)_ = 13.63, *p* = 0.0020], treatment [*F*_(1, 16)_ = 23.80, *p* = 0.00020]; interaction: [*F*_(1, 16)_ = 0.52, *p* = 0.48]) (Supplemental Fig. 9C). We found that the rats that consumed the WD exhibited reduced IL-33 concentration in plasma (diet: [*F*_(1, 16)_ = 8.67, *p* = 0.0095], treatment [*F*_(1, 16)_ = 2.70, *p* = 0.12]; interaction: [*F*_(1, 16)_ = 0.12, *p* = 0.74]) (Supplemental Fig. 9D). Supplemental Table 3 includes the results and statistics of the hippocampal cytokines tested in this study.

### WD consumption and exogenous NRG1 synergize to enhance hippocampal ErbB4 phosphorylation and sheddase protein levels

**Fig. 6:**
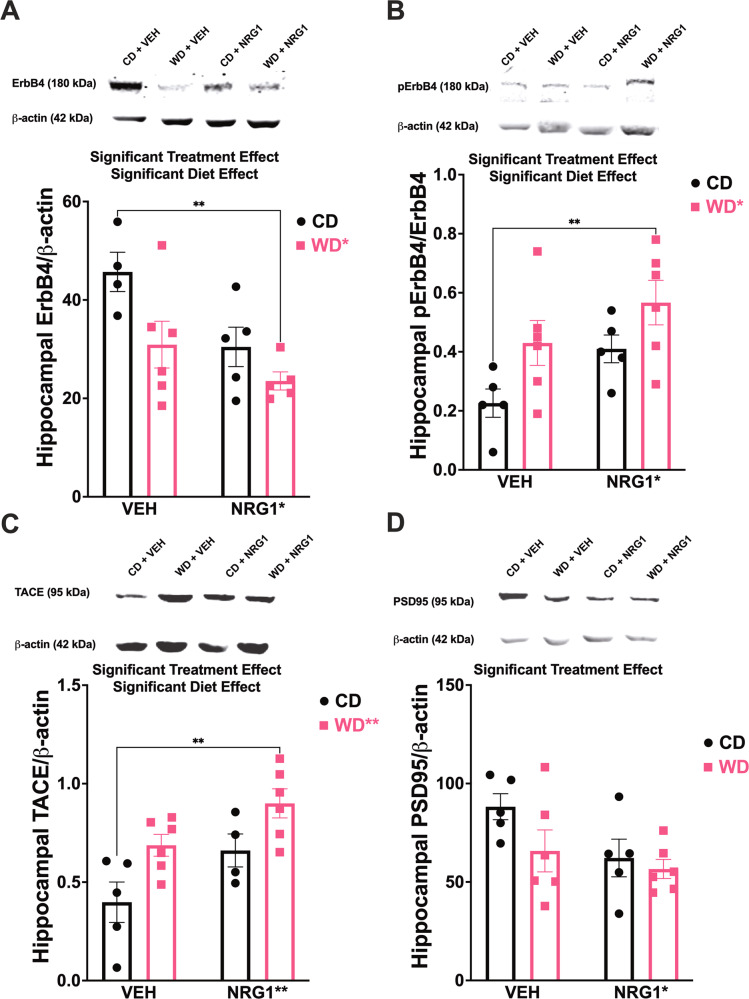
Obesogenic diet intake and subchronic NRG1 administration synergize to promote ErbB4 phosphorylation and TACE/ADAM17 protein levels in the hippocampus. **A** Obesogenic WD decreased total ErbB4 protein levels in the hippocampus (diet [*F*_(1, 16)_ = 7.34, *p* = 0.016]). WDN rats exhibited significantly lower ErbB4 protein levels when compared to CDV rats (*p* = 0.009). **B** Phosphorylation of hippocampal ErbB4 protein levels was affected by the treatment [*F*_(1, 18)_ = 5.82, *p* = 0.027] and diet [*F*_(1, 18)_ = 7.36, *p* = 0.014]. ErbB4 hyperphosphorylation was particularly evident in WDN rats relative to the CDV group (*p* = .012). **C** TACE/ADAM17 protein levels in the hippocampus were affected by the treatment [*F*_(1, 17)_ = 8.93, *p* = 0.008] and diet [*F*_(1, 17)_ = 11.03, *p* = 0.004]. WDN rats showed increased TACE/ADAM17 protein levels when compared to CDV rats (*p* = 0.001). **D** NRG1 administration showed a significant decrease for PSD-95 protein levels in the hippocampus (treatment [*F*_(1, 18)_ = 4.47, *p* = 0.048]). Sample size = 5–6 rats/group. Sample numbers: CD + VEH, *n* = 6; CD + NRG1, *n* = 6; WD + VEH, *n* = 6; WD + NRG1, *n* = 6.

ErbB4 is the predominant ErbB family member expressed in microglia [79] and has the highest affinity for NRGs, and was reported to bind to the NRG-1β isoform administered in this study avidly [80, 81]. ErbB3 binds NRG-1 but has very little kinase activity [82] and therefore must heterodimerize with other ErbB family members to activate signaling cascades. On the other hand, ErbB4 also differs from ErbB3 in that, when activated by NRGs, it is a highly active tyrosine kinase that can form functional receptor complexes by hetero- or homodimerization [83]. We and others have contributed to identifying the effects of NRG1-ErbB4 signaling on microglia/inflammation [37, 38, 79, 84–86]. Having established that NRG1-ErbB4 regulates inflammatory signals and microglia function, we focused on this family member [79, 87]. We found that the obesogenic WD decreased total ErbB4 protein levels in the hippocampus (Fig. 6A). Analyses revealed a significant treatment [*F*_(1, 16)_ = 7.97, *p* = 0.012] and diet [*F*_(1, 16)_ = 7.34, *p* = 0.016] effect, while showing no significant interaction [*F*_(1, 16)_ = 0.96, *p* = 0.34] effects on ErbB4 protein levels. Post hoc testing revealed that WDN rats exhibited significantly lower ErbB4 protein levels when compared to CDV rats (*p* = 0.009). Notably, exogenous NRG1 administration caused hyperphosphorylation of hippocampal ErbB4 (Fig. 6B). Analyses revealed a significant treatment [*F*_(1, 18)_ = 5.82, *p* = 0.027] and diet [*F*_(1, 18)_ = 7.36, *p* = 0.014] effect, while no significant interaction [*F*_(1, 18)_ = 0.13, *p* = 0.73] effects on protein levels. ErbB4 hyperphosphorylation was particularly evident in WDN rats relative to the CDV group (*p* = 0.012). Immunohistological evaluation demonstrated pErbB4 expression in hippocampal Cd11b/c cells from rats treated with exogenous NRG1 (Supplemental Fig. 6A). Supporting the expression and roles for NRG1-ErbB4 signaling in microglia cell function [38, 79, 85, 87].

NRG1 and ErbB4 (trans)activation via proteolytic processing is achieved by the shedding protease TNF-α-Converting Enzyme (*TACE*, also known as *ADAM17*) [88–90]. We found that the WD increased TACE/ADAM17 protein levels in the hippocampus (Fig. 6C). We identified a significant treatment [*F*_(1, 17)_ = 8.93, *p* = 0.008] and diet [*F*_(1, 17)_ = 11.03, *p* = 0.004] effect, while no significant interaction [*F*_(1, 17)_ = 0.099, *p* = 0.76] effects on protein levels. In agreement with the hippocampal pErbB4 protein levels results, we found that WDN rats showed increased TACE/ADAM17 protein levels when compared to CDV rats (*p* = 0.001).

We measured PSD-95, Akt, and Erk protein levels to investigate the impact of the WD and NRG1 on molecular pathways implicated in ErbB4 signaling. NRG1 administration showed a significant decrease for a postsynaptic regulator of ErbB4 dimerization, PSD-95 (Fig. 6D). Analyses revealed a significant treatment [*F*_(1, 18)_ = 4.47, *p* = 0.048], and no significant diet [*F*_(1, 18)_ = 2.83, *p* = 0.11] or interaction [*F*_(1, 18)_ = 1.02, *p* = 0.33] effects on PSD-95 protein levels. Raw Western blot images are included in Supplemental Figs. 10–13. Analyses for pAkt (normalized to total Akt) showed no significant interaction [*F*_(1, 17)_ = 0.71, *p* = 0.41], treatment [*F*_(1, 17)_ = 0.16, *p* = 0.69], or diet [*F*_(1, 17)_ = 2.80, *p* = 0.12] effects on protein levels (Supplemental Fig. 14A). Similarly, analyses for pErk1/2 (normalized to total Erk) showed no significant interaction [*F*_(1, 18)_ = 1.13, *p* = 0.30], treatment [*F*_(1, 18)_ = 0.88, *p* = 0.36], or diet [*F*_(1, 18)_ = 0.35, *p* = 0.56] effects on protein levels (Supplemental Fig. 14B).

### TACE/ADAM17 protein levels correlate with hippocampal pErbB4

We examined potential mechanisms explaining the reduced ErbB4 expression and increased pErbB4 protein levels in the rats that consumed the obesogenic WD. Twenty-one (21) days of consuming the WD and receiving NRG1 administration was not sufficient to significantly alter NRG1 protein levels in the hippocampus (Supplemental Fig. 15). Analyses showed no significant interaction [*F*_(1, 18)_ = 0.14, *p* = 0.72], treatment [*F*_(1, 18)_ = 0.031, *p* = 0.86], or diet [*F*_(1, 18)_ = 0.38, *p* = 0.54] effects on hippocampal NRG1 protein levels, as measured by ELISA.

Four (4) structurally and functionally distinct ErbB4 isoforms have been identified. One pair of isoforms differs within their extracellular juxtamembrane domains. These juxtamembrane ErbB4 isoforms are either susceptible (JMa) or resistant (JMb) to proteolytic processing that releases a soluble receptor ectodomain. We used qRT-PCR to examine the effect of the WD and NRG1 on the regulation of hippocampal ErbB4 isoforms. We found lower JMa mRNA levels in the WD rats (relative to CD rats) and higher JMb in the rats that received the exogenous NRG1 (relative to VEH) (Supplemental Fig. 16). Notably, mixed-effects model analyses revealed a significant interaction between isoform x diet [*F*_(3, 57)_ = 3.13, *p* = 0.033] and isoform x treatment [*F*_(3, 57)_ = 2.79, *p* = 0.049].

**Fig. 7:**
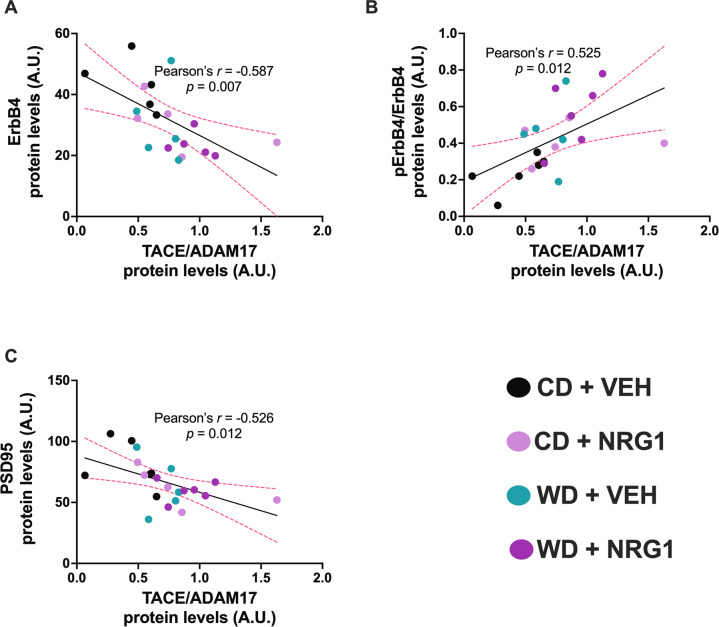
TACE/ADAM17 hippocampal protein levels are associated with ErbB4 activities. **A** ADAM17 hippocampal protein levels negatively correlates with ErbB4 protein levels (*r* = −0.59, *p* = 0.007). **B** ADAM17 protein levels positively correlates with hippocampal pErbB4 protein levels (*r* = 0.53, *p* = 0.012). **C** ADAM17 protein levels negatively correlates with PSD-95 protein levels in the hippocampus (*r* = −0.54, *p* = 0.012). Sample size = 5–6 rats/group. Sample numbers: CD + VEH, *n* = 6; CD + NRG1, *n* = 6; WD + VEH, *n* = 6; WD + NRG1, *n* = 6.

**Fig. 8:**
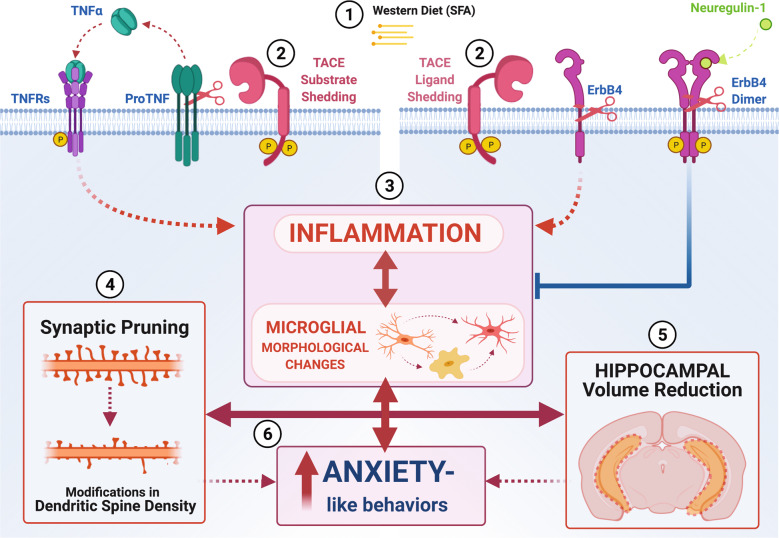
Working model unifying neurodevelopmental and neuroinflammatory hypotheses implicated in obesity-mediated hippocampal dysfunction. **1** It is plausible that exposure to obesogenic diets rich in saturated fatty acids (SFAs) increase TACE/ADAM17 sheddase expression and activities, **(2)** leading to dysregulation of neuroinflammatory (e.g., tumor necrosis factor alfa, TNF-α) and neurodevelopmental (ErbB4) mediators. **(3)** TNF receptor (TNFR) activation and prolonged ErbB4 cleavage tip the inflammatory balance to a pro-inflammatory state associated with microglial activation. **(4)** Neuroinflammation and microglial activation promote synaptic pruning and remodeling. **(5)** Disruption in microglia-synapse interactions contributes to synapse loss, dysfunction, and, consequently, hippocampal volume atrophy. **(6)** In summary, evidence in this study supports the conclusion that microglia respond to trophic factors and secrete cytokines, which are critical for normative synaptic plasticity in the maturing brain [137, 138]. Our data suggest that an imbalance in the levels of cytokines and neurotrophic factors during early pre-obesity states can impair microglia, synaptic plasticity, which may result in anxiety. Although our analyses focused on microglia and provided limited directionality of the effects, this dataset provides a rich resource for identifying meaningful synaptic targets in response to an obesogenic WD.

In summary, our data demonstrate that WD consumption reduces hippocampal ErbB4 protein levels while increasing its phosphorylation. TACE/ADAM17 activities may lead to reduced ErbB4 and also to ErbB4 transactivation [90]. We performed Pearson’s correlation analyses to test this idea. ADAM17 hippocampal protein levels showed a robust significant correlation with ErbB4 protein levels (*r* = −0.59, *p* = 0.007; Fig. 7A). ADAM17 protein levels were positively associated with hippocampal pErbB4 protein levels (*r* = 0.53, *p* = 0.012; Fig. 7B). A significant negative relationship was found between ADAM17 protein levels and PSD-95 protein levels in the hippocampus (*r* = −0.54, *p* = 0.012; Fig. 7C). Figure 8 describes our working model. We propose that obesogenic diets rich in saturated fatty acids promote TACE/ADAM17-mediated proteolytic cleavage of critical neurodevelopmental and neuroinflammatory processes, and consequently, lead to hippocampal synaptic/structural impairments implicated with anxiety disorders.

## Discussion

We designed the experiments to verify the thesis that exogenous NRG1 protects against obesity-related neuroinflammation, anxiety, and altered morphology of the rat hippocampus. We showed that exogenous NRG1 administration abolished the obesity-related overproduction of inflammatory cytokines in the hippocampus. NRG1 administration affected anxiety and hippocampal morphology independently to an obese-associated phenotype.

Obesity and the dietary intake of saturated fats and simple sugars have emerged as risk factors for the development of anxiety and stress-related disorders [2–6]. Consistent with studies in humans, we reported that the consumption of obesogenic diets in rats: (1) reduces hippocampal volume [18], (2) impairs the maturation of the corticolimbic fear circuits [48], (3) enhances behavioral vulnerabilities to psychosocial stress [18, 48, 91], (4) results in profound fear learning and extinction learning deficits [48, 91], even in the absence of an obesogenic phenotype [92], and (5) leads to alterations in biomarkers implicated in inflammatory responses [49]. As valuable as these earlier findings have been in constructing a conceptual framework for the impact of obesogenic environments on hippocampal maturation, our current understanding is primarily based on molecular, cellular, and behavioral analyses of obese versus control animals at the end of dietary manipulations (when rats are considered obese; in our model, approximately at eight weeks after WD exposure). Less is known about the early molecular events and mechanisms contributing to hippocampal structural and functional impairments in obesity, and thus was the focus of this study.

Here, we sought to determine whether exogenous NRG1 administration prevents early hippocampal abnormalities in adolescent rats exposed to an obesogenic Western diet (WD). We report that the obesogenic WD induces anxiety-like behaviors before the onset of obesity-related changes in metabolism. We also report unique anatomical vulnerabilities to the obesogenic WD, particularly in the CA1 subfield of the hippocampus. These behavioral and anatomical alterations were associated with changes in microglial morphology, unique neuroinflammatory cytokine profiles, ErbB4 hyperphosphorylation, and increased TACE/ADAM17 protein levels. Interestingly, the obesogenic WD-driven cytokine profiles were partly ameliorated in rats receiving exogenous NRG1. Exogenous NRG1 administration during adolescence altered hippocampal structure, microglia morphology, and the concentration of several inflammatory mediators. The main findings of the study are summarized in Supplemental Fig. 1.

### The consumption of an obesogenic diet during early adolescence promotes neuroinflammation and anxiety-like behaviors while increasing hemispheric differences in hippocampal volumes

Using a behavioral battery of tasks dependent on optimal hippocampal function, we demonstrated that three weeks on an obesogenic WD increased anxiety-like indices in the elevated plus maze in adolescent rats. The rats exposed to the obesogenic WD also exhibited increased locomotor activity (exploratory behavior) in the open field test. Trace fear conditioning and spatial working memory tasks were not affected in the rats that consumed the WD. An interesting finding of this study is that WD rats exhibited attenuated FPS reactivity during high-intensity tone trials (Supplemental Fig. 2). This failure to show enhanced startle reactivity during high-intensity tone trials could not be attributed to blunted reactivity to the aversive foot shocks or acoustic stimuli. Interestingly, this phenotype is strikingly consistent with our prior work on adolescent dietary obesity and traumatic stress [48]. We interpreted these observations as saturation of the fear circuitry, which is supported by studies by Walker and Davis [93].

Prior work from our group identified unique hippocampal structural vulnerabilities to obesogenic environments [18]. We reported that, during adolescence, the ventral hippocampus (vHPC) is highly susceptible to the impact of an obesogenic diet. In contrast, the dorsal hippocampus (dHPC) appears to be more vulnerable to stress in a psychogenic stress rat model [18]. Such selective vulnerability within the adolescent hippocampus implies region- and cell-specific molecular heterogeneity that remains poorly understood. Here, MRI-based volumetrics demonstrated that three weeks on the obesogenic diet was sufficient to enhance hippocampal lateralization, which is a structural signature associated with impaired cognition in humans [73]. Given that vHPC but not the dHPC CA1 fields project directly to hypothalamic nuclei critical in stress regulation, anxiety, and food intake control [16, 17, 94], we proposed that early structural alterations to this circuit represent a risk factor for both obesity-induced anxiety and anxiety-induced obesity. Animal studies have highlighted the role of the vHPC in the acquisition of mature brain physiology through the refinement of GABAergic circuits during adolescence.

GABAergic interneurons may represent a crucial cellular target in obesity. The calcium-binding protein parvalbumin (PV) is considered a core molecular marker for GABAergic interneurons. Interestingly, PV-positive cells circumscribed to the ventral region of the vHPC undergo protracted development and increase in numbers during mid-adolescence in rats [95]. Notably, these cells express high levels of ErbB4 [96], regulate spine formation [97], and are highly susceptible to environmental stressors due to their high metabolic activity [98]. Elegant studies by Reichelt et al., demonstrated that male mice that consume an obesogenic diet had reduced PV-positive cell numbers in the CA1 of the hippocampus [99]. These changes were associated with alterations in microglia morphology (less ramified, more ameboid) [99]. Notably, these effects were observed during adolescence and not during adulthood [99]. Collectively, these findings direct attention to the right vHPC CA1 as a relevant region in adolescent obesity and its related complications.

### Hyper-NRG1 conditions reduce hippocampal volume and attenuate trace fear conditioning and, in a diet-type dependent manner

Neuregulin-1 (NRG1) and its ErbB receptor tyrosine kinases are expressed in the developing nervous system and in the adult brain. ErbB4 is likely to be the primary mediator of NRG1 functions in the brain, and it is mainly expressed in hippocampal GABAergic interneurons, where it is enriched at postsynaptic terminals [100]. NRG1 signaling participates in several critical neurodevelopmental processes and is implicated in nerve cell differentiation and synapse formation, radial glia formation and neuronal migration, axon navigation, and neurite outgrowth [101]. Perturbations in NRG1/ErbB4 function have been associated with various neuropsychiatric disorders [101], resilience to stress [31], and obesity/metabolic syndrome [47, 57, 102, 103]. Emerging evidence suggests that ErbB4 may also be important in the pathogenesis of obesity in humans [24, 103].

The NRG1 isoform β that was administered in this study is more highly expressed in the brain and has the highest affinity for ErbB receptors [80, 81]. In the hippocampus, pro-NRG-1β is abundant in presynaptic CA3 pyramidal neurons, and it is cleaved and released in an activity-dependent manner [104–106]. Although exogenous NRG1 has a relatively short life in plasma, we and others have shown that NRG1 crosses the BBB [46, 107, 108]. A key finding of this study is that prolonged NRG1 administration during early adolescence reduced hippocampal volume in the rats that consumed the CD. The effects of NRG1 on hippocampal structure were associated with reduced ErbB4/PSD-95 protein levels in the hippocampus. ErbB4 is localized at the postsynaptic density (PSD) of glutamatergic synapses, presumably by interacting with the PSD protein PSD-95 [109–112]. PSD-95 expression promotes the maturation of excitatory (glutamatergic) synapses, increasing the number and size of dendritic spines [113]. Loss of synaptic connections is likely to have consequences for hippocampal network organization and volume [114, 115]. Interestingly, manipulating microglial activation with minocycline restores PSD-95 protein levels in the hippocampus of aged mice [116], which validates the role of these cells in regulating synaptic densities and hippocampal volume. It is also probable that the exogenous NRG1 acted peripherally to impact the brain. We have demonstrated that NRG1 influences several peripheral cytokines and inflammatory mediators (Supplemental Figure 9) [36]. Recent studies demonstrate that the fibroblast growth factor-21 (FGF21), an hepatokine, is partly responsible for the beneficial actions of an exogenously administered NRG1 fusion protein on obesity-related metabolic outcomes [47]. Here, WD rats exhibited higher FGF21 levels, particularly those receiving the exogenous NRG1 injections (Table 1), which supports the FGF-21-resistant state observed in obese humans [117]. Given that hippocampal microglia respond to FGF21 [118], exogenous NRG1 may alter microglial morphology and inflammatory mediators via FGF21 regulation. NRG1-ErbB4 dysregulation may result in altered inhibitory/excitatory (I/E) balance in hippocampal circuits and, thus, a limited ability to form and retain fear memories [119–121]. Together, it is clear that NRG1 is a potent modulator of hippocampal volume and cognition.

### Short-term exposure to an obesogenic WD and hyper-NRG1 conditions alter microglial morphology

Microglia cells, the resident immune cells in the brain, are responsible for maintaining a dynamic balance between anti-inflammatory and pro-inflammatory mediators. Microglia cells play a significant role in the behavioral outcomes associated with obesity [23, 122, 123]. However, the underlying molecular mechanisms regulating the microglia-dependent inflammatory balance remain poorly understood. Microglia express ErbB2, 3, and 4 receptors [84, 87, 124] and are highly responsive to NRG1 [38, 76]. Yet, the function of this signaling pathway within microglia is poorly understood. While it is very likely that the observed NRG1 effects are related to changes in local microcircuits (PV + interneurons exhibit high ErbB4 expression) [96], this study provides additional support to the notion that NRG1-ErbB4 signaling regulates microglial morphology and inflammatory mediators [38, 84–86, 125]. In our model, density, lacunarity, span ratio, and circularity were sensitive morphological indicators of WD- and NRG1-induced microglia changes. Evaluation of density (solidity) demonstrated that short-term exposure to an obesogenic WD significantly decreased this parameter in the right CA1 subfield. This decrease in density indicates a less compact phenotype. Microglial lacunarity reflects cellular heterogeneity and identifies sensitive changes in specific features (e.g., soma size relative to the length of processes) [78]. We found increased lacunarity in the right CA1 of WD rats, suggesting higher cellular complexity and perhaps a hyper-ramified transitional state relative to controls [126]. Together, the WD induced a morphotype usually accompanied by synaptic protein engulfment and remodeling [126–128]. Exogenous NRG1 administration resulted in a high span ratio (form factor) in the right CA1 region. The span ratio describes the cell shape and is based on the ratio of length and height of the Convex Hull area occupied by the cell [77]. The higher the span ratio, the more negligible area difference between the soma and processes, suggesting a more de-ramified/rod-like shape [129]. Interestingly, this phenotype is highly proliferative [130], which supports NRG1 effects on microglia [84]. Microglial circularity was also sensitive to exogenous NRG1. Circularity is a measure of shape and size and was found to be reduced in NRG1-treated rats. Low circularity values are associated with lower roundness (sphere-like) shapes and are inversely associated with the span ratio [129]. Taken together, adolescent exposure to an obesogenic WD and exogenous NRG1 influenced selective morphological metrics related to complexity, ramification, and shape. Our findings support the highly dynamic changes associated with microglia activation and synaptic remodeling [77, 126, 127].

### The interplay between neurotrophic and neuroinflammatory mediators in obesity

Genetic studies have demonstrated strong associations between single-nucleotide polymorphisms in the ErbB4 gene and obesity [24, 103]. Notably, while ErbB4 deletion predisposes mice to metabolic alterations implicated in obesity, NRG1 administration is known to improve metabolic health in obese mice by lowering blood glucose, improving insulin sensitivity, and reducing caloric intake [47, 57, 102]. Recent studies have reported a sex-specific interplay between NRG1 signaling and high-fat diet (HFD) consumption in mice [131–133]. Consistent with a growing list of studies demonstrating the potential involvement of NRG-ErbB signaling in obesity [24, 47, 57, 102, 103, 134], we found several significant interactions between the obesogenic diet and exogenous NRG1. We present new evidence demonstrating that exogenous NRG1 effects on the fear-potentiated startle, total hippocampal and CA1 subfield volume, and selective hippocampal cytokines (TNF-α, IL-10, IL-6) are influenced by short-term access to an obesogenic diet. These findings are in agreement with evidence supporting that neuroinflammation and palmitate can disrupt NRG1-ErbB4 signaling [39, 135]. Interestingly, exogenous NRG1 synergized with the obesogenic diet to enhance ErbB4 phosphorylation and its proteolytic processing [90]. ErbB4 proteolytic cleavage is mediated by the tumor necrosis factor-α-converting enzyme (TACE) / a Disintegrin And Metalloproteinase 17 (ADAM17)-dependent ectodomain shedding in a protein kinase C (PKC)-dependent manner. Here, we present data suggesting a role for TACE/ADAM17 on ErbB4 activities and synaptic integrity. Together with our previous studies, the findings reported here indicate that exposure to an obesogenic diet during adolescence selectively affects hippocampal structure and specific cognitive domains. Our investigations support that early adolescence is a critical window during which obesogenic diets may exert anxiogenic effects.

### Limitations

Our results demonstrate significant alterations in neurotrophic and neuroinflammatory signals in adolescent rats that consume an obesogenic diet enriched in saturated fats and simple sugars. However, the temporal window in which these effects take place was not established. The spatiotemporal expression of hippocampal ErbB4 and associated cytokine profiles must be determined to clarify a mechanism better. The impact of daily i.p. injections may confound the interpretation of the study. While this represents one of the best approaches to achieve therapeutic NRG1 levels in circulation (NRG1 has a short half-life), it is possible that the subchronic daily injection regime affected some endpoint measures. Furthermore, it is not clear from our results whether the NRG1 effects on hippocampal structure and behaviors are mediated by microglia, a change in the extracellular milieu via intermediate factors, or in response to intense neuronal activities induced by ErbB4 phosphorylation. Future studies should be carried out to disentangle the relative contribution of neurons and microglia to the observed effects. The use of inhibitors (e.g., AG1478) or fusion proteins (e.g., ecto-ErbB4) needs to be considered to dissect mechanisms and determine causality. Selecting appropriate single protein loading controls continues to represent a challenge for the Western blot technique, particularly for analyzing low-expressing protein targets in rodent models of diet-induced obesity. While the use of β-actin as loading control has been shown to be affected in mice models of obesity [136], the conditions of this study are different (e.g., diet composition, duration of exposure, rodent model, and age). However, in light of prior evidence, readers must exercise caution when interpreting molecular results normalized to most common housekeeping genes/proteins. Interpretation of the study may also be confounded by the effect of multiple behavioral tests on the same animal. Future experiments are required to dissociate the chronic vs. acute response to the NRG1 injection on molecular targets. Additionally, the study should be replicated in female rats and other rodent strains.

## Conclusion

Current pathophysiological models explaining how early exposure to obesogenic diets alters brain and behavior have focused on neurodevelopmental or immunological mechanisms. We report a novel interaction between these factors in the maturing hippocampus. Our results indicate that a short-term (21 days) dietary challenge with an obesogenic WD during the critical neurodevelopmental stage of adolescence disrupts ErbB4 activities in the hippocampus. Most importantly, we continue to provide evidence that WD consumption during adolescence leads to neuroanatomical, inflammatory, and molecular alterations related to depression, anxiety disorders, and cognitive impairments in humans. While extrapolating our data must be made carefully, our results indicate that TACE/ADAM17-ErbB4 hyperfunction may contribute to abnormal hippocampal structural and cognitive vulnerabilities in individuals who consume obesogenic diets.

## Supplementary information

Supplemental Material
