# Supplementary material for: Short-term exposure to an obesogenic diet during adolescence elicits anxiety-related behavior and neuroinflammation: modulatory effects of exogenous neuregulin-1
Source: Transl Psychiatry. 2022 Feb 26;12:83. doi: 10.1038/s41398-022-01788-2 (PMC8882169; doi:10.1038/s41398-022-01788-2)
Supplement: Supplementary file 1 — Supplemental Material [file 41398_2022_1788_MOESM1_ESM.docx]

**Vega-Torres et al., 2021_Short-term exposure to an obesogenic diet during adolescence elicits anxiety-related behavior and neuroinflammation: modulatory effects of exogenous neuregulin-1**

**Supplemental Materials**

**Supplemental Table 1. Macronutrient composition of the custom purified diets.** Detailed composition of the matched low-fat purified control diet (CD, 5-gm% fat, product *#F7463*) and Western-like high-saturated fat diet (WD, 20-gm% fat, product *#F7462*).

**Supplemental Table 2. List of antibodies.**

**Supplemental Table 3. Detailed cytokine profiles and related statistics.**

**Supplemental Table 4. Microglial descriptors and definitions.**

**Supplemental Table 5. Detailed microglial morphometric analyses values.**

**Supplemental Figure 1. Graphical abstract summarizing the main findings of this study.**

**Supplemental Figure 2. Obesogenic diet and prolonged NRG1 administration attenuate startle reactivity in a tone-dependent manner.** Each tone intensity from the trace conditioning protocol was analyzed (tone alone vs. light + tone; 90 dB [*F*_(1, 40)_ = 64.82, *p* < 0.0001], 95 dB [*F*_(1, 44)_ = 27.71, *p* < 0.0001], and 105 dB [*F*_(1, 43)_ = 37.61, *p* < 0.0001]) and only CDV rats exhibited significant differences between the stimulus type in all three tone intensities. Sample size = 12 rats / group.

**Supplemental Figure 3. Obesogenic diet increases ambulation and closed arm preference in a comprehensive behavioral test battery. (A)** Intake of an obesogenic diet during adolescence increased the time spent in the center of the open field maze (diet: [*F*_(1, 34)_ = 7.70, *p* = 0.009], treatment [*F*_(1, 34)_ = 0.17, *p* = 0.68]; interaction: [*F*_(1, 34)_ = 0.74, *p* = 0.40]) which was associated with increased locomotor activity. **(B)** The rats that consumed the obesogenic diet exhibited increased ambulation in the OFT (diet: [*F*_(1, 34)_ = 5.69, *p* = 0.023], treatment [*F*_(1, 34)_ = 0.00071, *p* = .0.98]; interaction: [*F*_(1, 34)_ = 0.024, *p* = 0.88]). **(C)** Duration in open arms was not affected by the diet or exogenous NRG1(diet: [*F*_(1, 42)_ = 0.30, *p* = 0.59], treatment [*F*_(1, 42)_ = 1.88, *p* = 0.18]; interaction: [*F*_(1, 42)_ = 0.035, *p* = 0.85]). **(D)** In contrast, the duration in the closed arms was significantly increased in the rats that consumed the obesogenic WD (diet: [*F*_(1, 42)_ = 17.10, *p* = 0.0002], treatment [*F*_(1, 42)_ = 0.28, *p* = 0.60]; interaction: [*F*_(1, 42)_ = 0.57, *p* = 0.45]. Spontaneous alternation is considered to reflect hippocampal-dependent short-term memory in rats.^1,2^ **(E)** In the Y-maze, spontaneous alternations (diet: [*F*_(1, 40)_ = 0.011, *p* = 0.92], treatment [*F*_(1, 40)_ = 0.97, *p* = 0.33]; interaction: [*F*_(1, 40)_ = 0.0074, *p* = 0.93]) and **(F)** the total number of alternations (diet: [*F*_(1, 40)_ = 0.22, *p* = 0.64], treatment [*F*_(1, 40)_ = 0.32, *p* = 0.58]; interaction: [*F*_(1, 40)_ = 1.00, *p* = 0.32]) were not affected by the experimental manipulations.

**Supplemental Figure 4. Total brain volume is not altered by short-term obesogenic WD and exogenous NRG1.** Total brain volume was not affect by the diet type [*F*_(1, 19)_ = 0.25, *p* = 0.62] or treatment [*F*_(1, 19)_ = 0.18, *p* = 0.67].

**Supplemental Figure 5. Obesogenic diet increases hippocampal lateralization.** Hippocampal volumes were significantly affected by the diet [*F*_(1, 19)_ = 4.71, *p* = 0.043] in a hemisphere-specific manner [lateralization effect: *F*_(1, 19)_ = 6.08, *p* = 0.023] and. **(A-D)** Only the WD VEH group **(C)** exhibited a significantly reduced right hippocampal volume relative to the left hippocampal volume (*t*_(5)_ = 2.66, *p* = 0.045). Circled data illustrates a significant outlier. Removing this animal from the analysis did not alter the significance of the t-test and interpretation of the result. Sample size = 6 rats / group.

**Supplemental Figure 6. (A) pErbB4 is expressed in macrophages/microglia in the hippocampus of rats treated with NRG1. (B)** Representative Iba-1-stained section from CA1 region of WD NRG1 rat. **(C)** Representative section from CA1 region of WD NRG1 rat were Iba-1 antibody was omitted. Nonspecific binding control validates the specificity of the commercial anti-Iba-1 primary antibody. Scale bars: A, 20 microns; B-C, 100 microns.

**Supplemental Figure 7. PC analysis reveals distinctive microglial morphological descriptors in each group. (A-D)** Principal Component (PC) analysis on the morphometric parameters to trace the possible differences in microglia driving the changes in hippocampal structure and behavior. These PCs explained more than 80% of the accumulated variance between cells (PC1: ~60%; PC2: ~20%; PC3: ~5%). Sample size approximately 500 microglia / group.

**Supplemental Figure 8. PCA reveals distinctive microglial morphological descriptors contributing to differences between groups.** Seven morphometric parameters describing microglial shape were subjected to PCA and there were distinctive morphological profiles between groups (PC1 = 58, PC2 = 35%). Sample size = 3 rats / group.

**Supplemental Figure 9. Short-term exposure to an obesogenic WD and exogenous NRG1 elicits selective alterations in circulating cytokine/chemokine concentrations. (A)** Exogenous NRG1 decreased IL-10 plasma concentration (diet: [*F*_(1, 16)_ = 2.38, *p* = 0.14], treatment [*F*_(1, 16)_ = 15.71, *p* = 0.0011]; interaction: [*F*_(1, 16)_ = 0.17, *p* = 0.69]). **(B)** Similarly, exogenous NRG1 administration reduced IL-17A concentration in plasma (diet: [*F*_(1, 15)_ = 0.57, *p* = 0.46], treatment [*F*_(1, 15)_ = 7.18, *p* = 0.017]; interaction: [*F*_(1, 15)_ = 2.02, *p* = 0.18]). **(C)** Interestingly, we found that while the obesogenic WD increased the concentration of CXCL1, exogenous NRG1 resulted in a marked reduction in the levels of this important chemokine (diet: [*F*_(1, 16)_ = 13.63, *p* = 0.0020], treatment [*F*_(1, 16)_ = 23.80, *p* = 0.00020]; interaction: [*F*_(1, 16)_ = 0.52, *p* = 0.48]). **(D)** The rats that consumed the obesogenic WD exhibited reduced IL-33 concentration in plasma (diet: [*F*_(1, 16)_ = 8.67, *p* = 0.0095], treatment [*F*_(1, 16)_ = 2.70, *p* = 0.12]; interaction: [*F*_(1, 16)_ = 0.12, *p* = 0.74]).

**Supplemental Figures 10-13.** Western blot images used for densitometric analyses. Please note that images were subjected to background subtraction after the blots were quantified. Final images are included in Figure 6.

**Supplemental Figure 10. Western blot images illustrating the complete sample set used for ErbB4 detection and analyses.**

**Supplemental Figure 11. Western blot images illustrating the complete sample set used for pErbB4 detection and analyses.**

**Supplemental Figure 12. Western blot images illustrating the complete sample set used for TACE/ADAM17 detection and analyses.**

**Supplemental Figure 13. Western blot images illustrating the complete sample set used for PSD95 detection and analyses.**

**Supplemental Figure 14. ErbB4 downstream mediators are not affected significantly. (A)** pAkt levels (normalized to total Akt) were not affected by the treatment [*F*_(1, 17)_ = 0.16, *p* = 0.69] or diet [*F*_(1, 17)_ = 2.80, *p* = 0.12]. **(B)** pErk1/2 levels (normalized to total Erk) were not affected by the treatment [*F*_(1, 18)_ = 0.88, *p* = 0.36] or diet [*F*_(1, 18)_ = 0.35, *p* = 0.56]. Sample size = 5-6 rats / group.

**Supplemental Figure 15. NRG1 levels are not significantly altered.** Twenty-one (21) days consuming the WD (diet [*F*_(1, 18)_ = 0.38, *p* = 0.54]) and receiving NRG1 (treatment [*F*_(1, 18)_ = 0.031, *p* = 0.86]) administration was not sufficient to significantly alter NRG1 protein levels in the hippocampus, as measured by ELISA. Sample size = 5-6 rats / group.

**Supplemental Figure 16. Obesogenic diet and prolonged NRG1 treatment alter ErbB4 isoforms expression in the hippocampus.** A subset of rats from Study 1 was used for qPCR analyses. The rats were euthanized with Euthasol (Virbac) and briefly perfused with PBS. The rats were rapidly decapitated, left hippocampal tissue harvested, and tissue immediately submerged in RNAlater solution to preserve RNA quality and quantity. The cDNA was amplified by PCR using the following primer sets (5’-3’):

*Gapdh*, fwd: AGTTCAACGGCACAGTCAAG,

*Gapdh*, rev: GTGGTGAAGACGCCAGTAGA;

*ErbB4-JMa*, fwd: GGACGGGCCATTCCACTTTACC,

*ErbB4-JMa*, rev: CCTCCAATGACTCCGGCTGC;

*ErbB4-cyt1*, fwd: GGAATATTTGGTCCCCCAGGCTTTC,

*ErbB4-cyt1*, rev: GAGGAGGGCTGTGTCCAATTTCAC;

*ErbB4-JMb*, fwd: CATTGAAGACTGCATCGGCCTG,

*ErbB4-JMb*, rev: CCTCCAATGACTCCGGCTGC;

*ErbB4-cyt2*, fwd: GGAATATTTGGTCCCCCAGGCTTTC,

*ErbB4-cyt2*, rev: GTACACAAACTGATTCCTATTGGAGTCAATTC.

The qRT-PCR methods have been detailed in previous publication from our laboratory ^3^. Mixed-effects model analyses revealed a significant interaction between isoform x diet [*F*_(3, 57)_ = 3.13, *p* = 0.033] and isoform x treatment [*F*_(3, 57)_ = 2.79, *p* = 0.049]. Analyses demonstrated lower JMa mRNA levels in the WD rats (relative to CD rats) and higher JMb in the rats that received the exogenous NRG1 (relative to VEH). Sample size = 6 rats / group (before detecting outliers).

**Replication Statement**

Four animal cohorts were used in this study. Behavioral testing and MRI experiments were performed once. Histological and molecular experiments were performed at least twice (per assay/per sample).

**Supplemental Table 1. Macronutrient composition of the custom purified diets.**

|  | **CD** | **WD** |
| --- | --- | --- |
| **Macronutrient (main source)** | **% *kcal*** | **% *kcal*** |
| Carbohydrates (corn starch) | 64.7 | 43.1 |
| Protein (casein) | 18.8 | 15.5 |
| Fat (milk fat) | 16.5 | 41.4 |
| ***Total kcal*** | 3.77 | 4.57 |
|  |  |  |
| **Fatty Acid Class** | **g/kg Diet** | **g/kg Diet** |
| Butyric (C4:0) | 0.72 | 5.10 |
| Caproic (C6:0) | 0.52 | 3.55 |
| Caprylic (C8:0) | 0.33 | 2.21 |
| Capric (C10:0) | 0.78 | 5.35 |
| Lauric (C12:0) | 0.93 | 6.30 |
| Myristic (C14:0) | 3.00 | 20.3 |
| Myristoleic (C14:1) | 0.24 | 1.67 |
| Pentadecanoic (C15:0) | 0.33 | 2.18 |
| Palmitic (C16:0) | 11.6 | 61.6 |
| Palmitoleic (C16:1) | 0.46 | 2.84 |
| Heptadecanoic (17:0) | 0.17 | 1.04 |
| Stearic (C18:0) | 3.52 | 20.2 |
| Oleic (C18:1) | 11.2 | 39.8 |
| Linoleic (C18:2) | 11.5 | 10.5 |
| Alpha Linolenic (C18:3) | 0.31 | 0.83 |
| Arachidic (C20:0) | 0.11 | 0.28 |
| Homogamma Linolenic (C20:3) | <0.07 | 0.20 |
| Arachidonic (C20:4 n-6) | <0.07 | 0.30 |
| EPA (C20:5 n-3) | <0.07 | <0.14 |
| DHA (C22:6 n-3) | <0.07 | <0.14 |
|  |  |  |
| **Fatty Acid Class** | **g/kg Diet** | **g/kg Diet** |
| Saturated | 20.8 | 121 |
| Monosaturated | 12.7 | 492 |
| Polyunsaturated | 11.3 | 11.5 |
| Omega-3 Fatty Acids | 0.31 | 0.94 |
| Omega-6 Fatty Acids | 11.5 | 10.1 |

**Supplemental Table 2. List of antibodies.**

| **Target** | **Company (Product)** | **Dilution** |
| --- | --- | --- |
| ADAM17 | Abcam (ab13535) | 1:2500 |
| Akt | Cell Signaling (2920) | 1:200 |
| β-actin | LI-COR (926-42212) | 1:2000 |
| EGFR (ErbB1) | Invitrogen (PA1-1110) | 1:500 |
| ErbB2 | Invitrogen (MA5-13105) | 1.5 𝜇g/mL |
| ErbB3 | Invitrogen (MA5-12675) | 3 𝜇g/mL |
| ErbB4 | Abcam (ab32375) | 1:1000 |
| Iba-1 | Wako (019-19741) | 1:750 |
| NRG1 | Invitrogen (MA5-12896) | 2 𝜇g/mL |
| p44/42 MAPK (Erk1/2) | Cell Signaling (9102) | 1:1000 |
| Phospho-Akt | Cell Signaling (2965) | 1:1000 |
| Phospho-ErbB4 (Y1284) | Abcam (ab61059) | 1:1000 |
| Phospho-p44/42 MAPK (Erk1/2) (Thr202/Tyr204) | Cell Signaling (9101) | 1:1000 |

**Supplemental Table 3. Detailed cytokine profiles and related statistics.**

| **Cytokines** | **Mean ± Standard Error of the Mean (pg/mg)** | | | | ***F*** | ***p*** |
| --- | --- | --- | --- | --- | --- | --- |
|  | **Control Diet** | | **Western Diet** | |  |  |
|  | **Vehicle** | **NRG-1** | **Vehicle** | **NRG-1** |  |  |
| IL-10 | 8.44 ± 0.96 | 19.90 ± 3.38 | 35.14 ± 9.71 | 20.56 ± 3.99 | Interaction, 4.683 | **Interaction, 0.0441** |
|  |  |  |  |  | Drug, 0.06692 | Drug, 0.7988 |
|  |  |  |  |  | Diet, 5.171 | **Diet, 0.0354** |
| TNF-α | 2.15 ± 0.10 | 4.61 ± 0.46 | 8.81 ± 2.04 | 4.54 ± 0.75 | Interaction, 7.620 | **Interaction, 0.0129** |
|  |  |  |  |  | Drug, 0.5545 | Drug, 0.4661 |
|  |  |  |  |  | Diet, 7.285 | **Diet, 0.0147** |
| IL-1α | 1.89 ± 0.23 | 2.40 ± 0.35 | 3.94 ± 0.66 | 2.78 ± 0.42 | Interaction, 3.542 | Interaction, 0.0771 |
|  |  |  |  |  | Drug, 0.5266 | Drug, 0.4779 |
|  |  |  |  |  | Diet, 7.502 | **Diet, 0.0140** |
| IL-1β | 26.04 ± 3.51 | 33.64 ± 2.17 | 51.36 ± 11.07 | 36.74 ± 6.16 | Interaction, 2.351 | Interaction, 0.1426 |
|  |  |  |  |  | Drug, 0.2340 | Drug, 0.6344 |
|  |  |  |  |  | Diet, 3.846 | Diet, 0.0655 |
| IL-6 | 193.63 ± 20.64 | 301.67 ± 33.35 | 354.98 ± 41.54 | 266.54 ± 34.69 | Interaction, 7.988 | **Interaction, 0.0112** |
|  |  |  |  |  | Drug, 0.07946 | Drug, 0.7812 |
|  |  |  |  |  | Diet, 3.297 | Diet, 0.0861 |
| IFN-γ | 66.65 ± 18.03 | 47.93 ± 7.76 | 64.12 ± 8.19 | 31.96 ± 7.76 | Interaction, 0.3843 | Interaction, 0.5431 |
|  |  |  |  |  | Drug, 5.514 | **Drug, 0.0305** |
|  |  |  |  |  | Diet, 0.7291 | Diet, 0.4044 |
| CXCL1 | 152.59 ± 3.88 | 145.80 ± 9.21 | 165.27 ± 16.81 | 126.12 ± 11.40 | Interaction, 1.788 | Interaction, 0.1978 |
|  |  |  |  |  | Drug, 3.603 | Drug, 0.0738 |
|  |  |  |  |  | Diet, 0.08352 | Diet, 0.7759 |
| CCL-2 | 174.48 ± 8.05 | 136.63 ± 13.80 | 170.40 ± 9.62 | 153.06 ± 7.60 | Interaction, 1.074 | Interaction, 0.3137 |
|  |  |  |  |  | Drug, 7.773 | **Drug, 0.0121** |
|  |  |  |  |  | Diet, 0.3892 | Diet, 0.5405 |
| GM-CSF | 33.02 ± 4.32 | 27.02 ± 5.73 | 53.48 ± 4.13 | 29.90 ± 7.60 | Interaction, 2.209 | Interaction, 0.1555 |
|  |  |  |  |  | Drug, 6.250 | **Drug, 0.0229** |
|  |  |  |  |  | Diet, 3.888 | Diet, 0.0651 |
| IL-18 | 1,056.79 ± 57.36 | 1,074.40 ± 68.86 | 1,102.42 ± 36.09 | 1,106.25 ± 60.97 | Interaction, 0.01497 | Interaction, 0.9040 |
|  |  |  |  |  | Drug, 0.03624 | Drug, 0.8512 |
|  |  |  |  |  | Diet, 0.4736 | Diet, 0.5001 |
| IL-12p70 | 82.11 ± 4.80 | 91.07 ± 7.73 | 105.17 ± 10.55 | 88.31 ± 14.19 | Interaction, 1.482 | Interaction, 0.2392 |
|  |  |  |  |  | Drug, 0.1385 | Drug, 0.7141 |
|  |  |  |  |  | Diet, 0.9161 | Diet, 0.3512 |
| IL-17a | 18.83 ± 0.54 | 19.17 ± 1.54 | 20.69 ± 1.96 | 15.69 ± 2.39 | Interaction, 2.062 | Interaction, 0.1682 |
|  |  |  |  |  | Drug, 1.570 | Drug, 0.2263 |
|  |  |  |  |  | Diet, 0.1897 | Diet, 0.6683 |
| IL-33 | 357.80 ± 38.71 | 328.15 ± 33.89 | 370.01 ± 27.68 | 255.37 ± 29.44 | Interaction, 1.717 | Interaction, 0.2074 |
|  |  |  |  |  | Drug, 4.950 | **Drug, 0.0399** |
|  |  |  |  |  | Diet, 0.8724 | Diet, 0.3634 |

**Supplemental Table 4. Summary of Microglial Morphological Parameters**

| **Parameters** | **Measure** | **Unit** |
| --- | --- | --- |
| Objects | Cell number | *Cell* |
| Density | $\frac{\# of pixels within cell outline}{Convex Hull Area}$ | $\frac{\# of pixels}{{pixels}^{2}}$ |
| Span Ratio | $\frac{\boldsymbol{Convex Hull Elipse Longest Lenght}}{\boldsymbol{Convex Hull Elipse Longest Width}}$ | *Ratio (a.u)* |
| Maximum Span Across Hull | Maximum distance between two points across the Convex Hull | *pixels* |
| Convex Hull Area | Area of the polygon containing the whole cell shape | *pixels^2^* |
| Perimeter | Length of the single outline of the cell shape | *pixels* |
| Circularity | $\frac{\boldsymbol{4}\boldsymbol{\pi(cell area)}}{\left( \boldsymbol{cell perimeter} \right)^{\boldsymbol{2}}}$ | *pixels* |
| Width of Bounding Rectangle | Length of the width of the largest bounding rectangle | *pixels* |
| Height of Bounding Rectangle | Length of the height of the largest bounding rectangle | *pixels* |
| Max Radius from Hull's Centre of Mass | Radius of the largest circle bounding the convex hull area | *pixels* |
| Max/Min Radii | $\frac{Radius of the largest bounding circle}{radius of the smallest bounding circle}$ | *Radio (a.u)* |
| Mean Radius | $\frac{Max Radius+Min radius}{2}$ | *pixels* |
| Diameter of Bounding Circle | Diameter of the largest circle bounding the cell | *pixels* |
| Maximum Radius | Radius of the largest circle bounding the cell | *pixels* |
| CV for all Radii from Circle's Centre | Coeficient of variability for all the radii of all the circles bounding the cell | *Index of variability (a.u)* |
| CV for all Radii | Coeficient of variability for all the radii of all the circles bounding the convex hull area | *Index of variability (a.u)* |
| Mean Radii | Average of all radii from all bounding circles within the convex hull area | *pixels* |
| Lacunarity (Л) | Coefficient of variation expressed as pixel density per box as a function of box size | *Index of variability (a.u)* |

*a.u = arbitrary units*

**Table 5. Summary of Microglial Morphological Parameters Values**

| **Parameters** | **Mean ± Standard Error of the Mean** | | | | | | | | | | | | | | | |
| --- | --- | --- | --- | --- | --- | --- | --- | --- | --- | --- | --- | --- | --- | --- | --- | --- |
|  | **Control Diet** | | | | | | | | **Western Diet** | | | | | | | |
|  | **Vehicle** | | | | **NRG1** | | | | **Vehicle** | | | | **NRG1** | | | |
|  | **Left** | | **Right** | | **Left** | | **Right** | | **Left** | | **Right** | | **Left** | | **Right** | |
|  | **Dorsal** | **Ventral** | **Dorsal** | **Ventral** | **Dorsal** | **Ventral** | **Dorsal** | **Ventral** | **Dorsal** | **Ventral** | **Dorsal** | **Ventral** | **Dorsal** | **Ventral** | **Dorsal** | **Ventral** |
| Objects | 12.11 ± 0.56 | 13.22 ± 3.11 | 12.44 ± 3.18 | 11.89 ± 4.59 | 16.00 ± 3.61 | 15.00 ± 0.51 | 13.22 ± 1.68 | 12.22 ± 0.62 | 14.22 ± 0.29 | 14.56 ± 1.64 | 17.22 ± 1.49 | 15.44 ± 0.87 | 15.22 ± 2.04 | 17.22 ± 3.35 | 10.22 ± 2.25 | 10.44 ± 0.62 |
| Density | 0.57 ± 0.16 | 0.43 ± 0.14 | 0.40 ± 0.06 | 0.50 ± 0.11 | 0.29 ± 0.02 | 0.32 ± 0.02 | 0.36 ± 0.06 | 0.38 ± 0.06 | 0.27 ± 0.03 | 0.27 ± 0.03 | 0.26 ± 0.01 | 0.21 ± 0.02 | 0.34 ± 0.08 | 0.32 ± 0.07 | 0.28 ± 0.04 | 0.34 ± 0.06 |
| **Span Ratio** | **2.10 ± 0.21** | **1.88 ± 0.03** | **2.02 ± 0.02** | **1.77 ± 0.10** | **1.80 ± 0.07** | **1.97 ± 0.06** | **1.95 ± 0.10** | **2.09 ± 0.01** | **1.79 ± 0.10** | **1.80 ± 0.01** | **1.91 ± 0.06** | **1.83 ± 0.05** | **2.04 ± 0.07** | **2.01 ± 0.08** | **1.92 ± 0.01** | **2.29 ± 0.05** |
| **Maximum Span Across Hull** | **86.21 ± 23.79** | **114.09 ± 33.30** | **115.05 ± 16.39** | **94.37 ± 30.57** | **151.39 ± 20.61** | **138.34 ± 18.78** | **130.72 ± 32.07** | **118.28 ± 23.03** | **165.78 ± 30.38** | **159.78 ± 14.18** | **151.94 ± 3.69** | **174.55 ± 14.69** | **131.27 ± 28.21** | **134.50 ± 22.29** | **144.76 ± 14.01** | **130.19 ± 18.35** |
| **Convex Hull Area** | **3,366 ± 1,658** | **6,443 ± 3,530** | **5,707 ± 1,530** | **4,598 ± 2,894** | **10,482 ± 3,564** | **8,304 ± 2,481** | **8,237 ± 4,523** | **6,366 ± 2,358** | **13,321 ± 5,130** | **12,095 ± 2,678** | **10,262 ± 1,195** | **13,412 ± 2,377** | **7,597 ± 3,189** | **7,566 ± 2,192** | **8,343 ± 1,304** | **6,478 ± 1,616** |
| Perimeter | 212.76 ± 57.46 | 286.98 ± 86.04 | 285.26 ± 41.98 | 239.02 ± 77.45 | 384.77 ± 56.46 | 347.12 ± 47.86 | 328.20 ± 86.28 | 294.37 ± 59.18 | 423.51 ± 81.64 | 407.88 ± 38.47 | 385.42 ± 15.07 | 444.27 ± 42.51 | 326.49 ± 73.00 | 335.30 ± 55.93 | 362.21 ± 35.11 | 316.88 ± 44.33 |
| **Circularity** | **0.714 ± 0.046** | **0.729 ± 0.005** | **0.716 ± 0.003** | **0.759 ± 0.028** | **0.732 ± 0.020** | **0.713 ± 0.005** | **0.717 ± 0.017** | **0.709 ± 0.004** | **0.742 ± 0.025** | **0.735 ± 0.014** | **0.724 ± 0.013** | **0.738 ± 0.015** | **0.703 ± 0.009** | **0.710 ± 0.005** | **0.712 ± 0.004** | **0.679 ± 0.005** |
| Width of Bounding Rectangle | 70.44 ± 19.03 | 89.10 ± 20.98 | 92.09 ± 13.91 | 71.11 ± 24.65 | 122.23 ± 19.01 | 105.38 ± 14.93 | 107.02 ± 28.05 | 93.41 ± 19.36 | 137.67 ± 27.18 | 126.62 ± 12.79 | 118.88 ± 3.93 | 139.26 ± 10.98 | 101.24 ± 22.97 | 100.18 ± 13.13 | 110.54 ± 9.17 | 94.03 ± 10.09 |
| Height of Bounding Rectangle | 66.05 ± 17.26 | 95.11 ± 33.46 | 91.64 ± 13.17 | 82.32 ± 24.75 | 124.28 ± 16.62 | 116.00 ± 15.29 | 103.58 ± 27.18 | 93.51 ± 17.91 | 133.68 ± 26.34 | 133.54 ± 12.84 | 125.03 ± 7.37 | 144.28 ± 15.30 | 106.17 ± 23.40 | 112.77 ± 20.55 | 120.90 ± 13.66 | 108.77 ± 17.67 |
| Max Radius from Hull's Centre of Mass | 48.56 ± 11.78 | 62.77 ± 17.67 | 63.38 ± 9.09 | 52.45 ± 16.06 | 82.28 ± 10.78 | 75.39 ± 9.62 | 71.70 ± 16.86 | 64.15 ± 11.97 | 89.35 ± 15.57 | 86.65 ± 7.20 | 82.67 ± 2.05 | 94.41 ± 7.75 | 71.73 ± 15.01 | 73.53 ± 11.23 | 79.72 ± 7.89 | 71.11 ± 9.52 |
| Max/Min Radii | 2.60 ± 0.21 | 2.08 ± 0.13 | 2.20 ± 0.08 | 1.99 ± 0.09 | 1.94 ± 0.07 | 2.01 ± 0.03 | 2.03 ± 0.13 | 2.13 ± 0.04 | 1.88 ± 0.09 | 1.94 ± 0.07 | 1.94 ± 0.05 | 1.92 ± 0.13 | 2.02 ± 0.11 | 2.02 ± 0.06 | 2.05 ± 0.06 | 2.38 ± 0.09 |
| Mean Radius | 37.62 ± 9.97 | 50.09 ± 14.62 | 50.04 ± 7.47 | 41.26 ± 13.33 | 65.77 ± 9.33 | 60.49 ± 8.34 | 57.44 ± 14.70 | 51.40 ± 10.08 | 73.03 ± 13.38 | 70.67 ± 6.31 | 66.89 ± 2.28 | 76.49 ± 7.35 | 56.97 ± 12.48 | 58.92 ± 9.47 | 63.11 ± 6.16 | 56.15 ± 7.88 |
| Diameter of Bounding Circle | 86.82 ± 23.97 | 115.05 ± 33.67 | 115.75 ± 16.63 | 95.10 ± 30.98 | 152.96 ± 20.64 | 139.48 ± 18.92 | 131.68 ± 32.30 | 119.32 ± 23.33 | 167.14 ± 30.52 | 161.29 ± 14.16 | 153.11 ± 3.98 | 175.89 ± 15.06 | 132.44 ± 28.45 | 135.55 ± 22.43 | 145.73 ± 13.99 | 131.04 ± 18.30 |
| Maximum Radius | 43.41 ± 11.99 | 57.53 ± 16.84 | 57.87 ± 8.31 | 47.55 ± 15.49 | 76.48 ± 10.32 | 69.74 ± 9.46 | 65.84 ± 16.15 | 59.66 ± 11.66 | 83.57 ± 15.26 | 80.65 ± 7.08 | 76.56 ± 1.99 | 87.94 ± 7.53 | 66.22 ± 14.23 | 67.78 ± 11.21 | 72.87 ± 7.00 | 65.52 ± 9.15 |
| CV for all Radii from Circle's Centre | 0.17 ± 0.01 | 0.16 ± 0.01 | 0.18 ± 0.01 | 0.16 ± 0.00 | 0.17 ± 0.01 | 0.17 ± 0.00 | 0.16 ± 0.01 | 0.18 ± 0.00 | 0.15 ± 0.01 | 0.15 ± 0.01 | 0.16 ± 0.01 | 0.17 ± 0.02 | 0.17 ± 0.01 | 0.16 ± 0.01 | 0.17 ± 0.01 | 0.19 ± 0.01 |
| CV for all Radii | 0.24 ± 0.02 | 0.21 ± 0.01 | 0.21 ± 0.01 | 0.20 ± 0.01 | 0.19 ± 0.01 | 0.19 ± 0.00 | 0.20 ± 0.02 | 0.21 ± 0.01 | 0.17 ± 0.01 | 0.18 ± 0.01 | 0.19 ± 0.01 | 0.18 ± 0.02 | 0.21 ± 0.01 | 0.19 ± 0.01 | 0.20 ± 0.01 | 0.23 ± 0.01 |
| Mean Radii | 37.85 ± 10.57 | 50.35 ± 14.90 | 50.31 ± 7.59 | 41.40 ± 13.53 | 66.43 ± 9.49 | 61.16 ± 8.40 | 57.94 ± 14.92 | 51.80 ± 10.36 | 73.69 ± 13.39 | 71.55 ± 6.44 | 67.83 ± 2.20 | 77.50 ± 7.46 | 57.66 ± 12.71 | 59.75 ± 9.79 | 64.08 ± 6.62 | 56.54 ± 8.13 |
| Lacunarity (Л) | 0.31 ± 0.07 | 0.39 ± 0.06 | 0.40 ± 0.03 | 0.36 ± 0.05 | 0.44 ± 0.02 | 0.41 ± 0.01 | 0.40 ± 0.03 | 0.40 ± 0.03 | 0.51 ± 0.06 | 0.45 ± 0.01 | 0.46 ± 0.01 | 0.55 ± 0.03 | 0.42 ± 0.04 | 0.43 ± 0.03 | 0.44 ± 0.02 | 0.43 ± 0.04 |

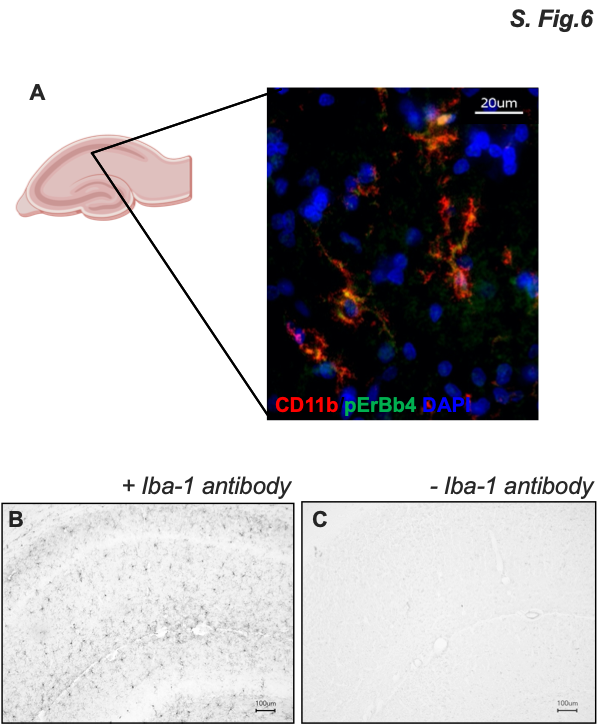

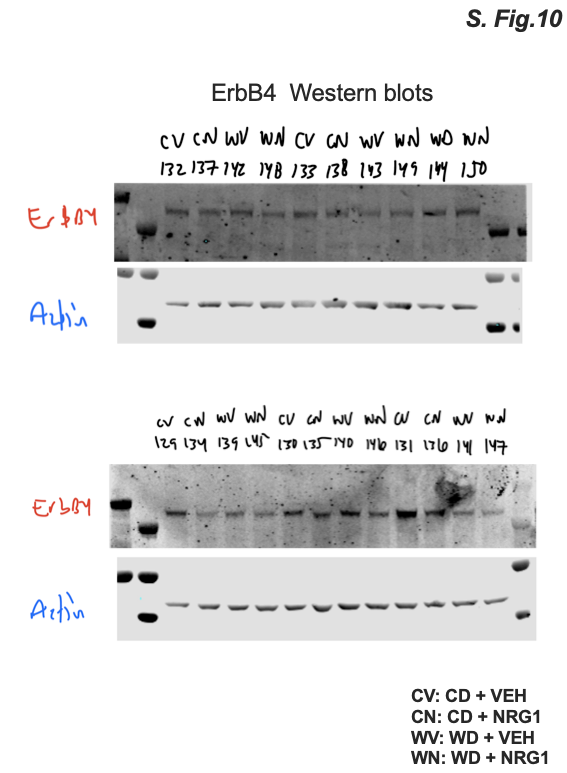


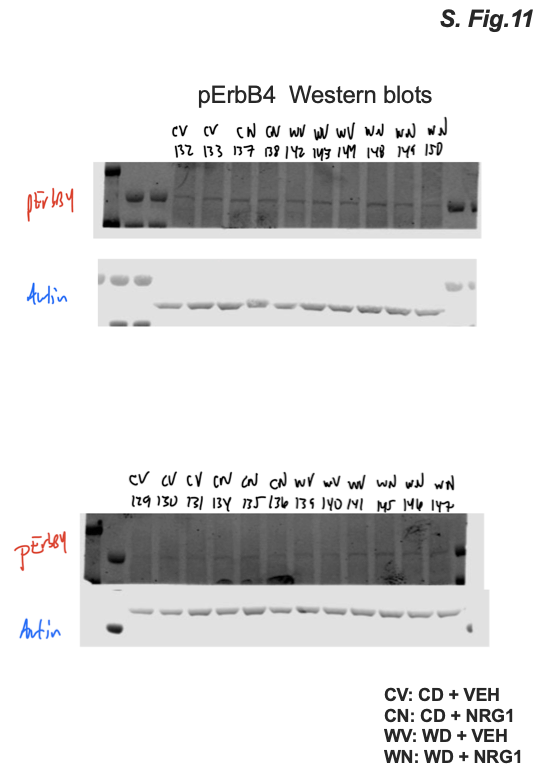


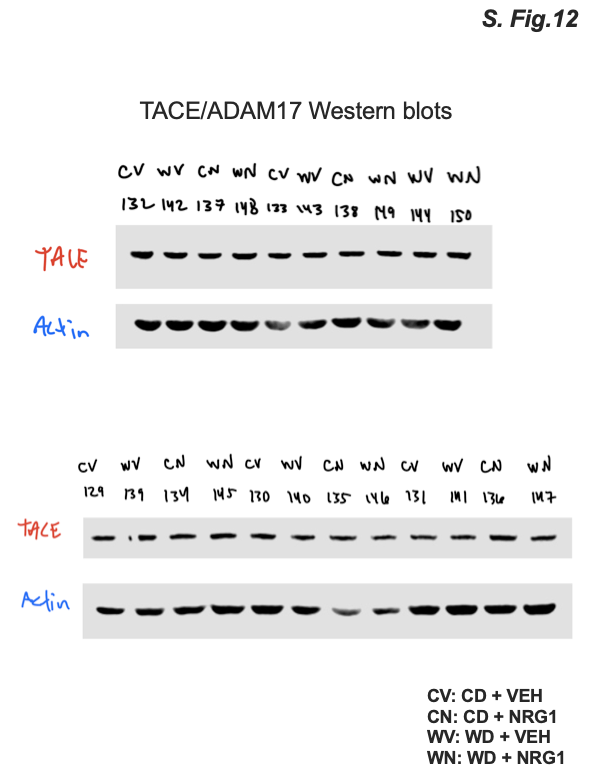


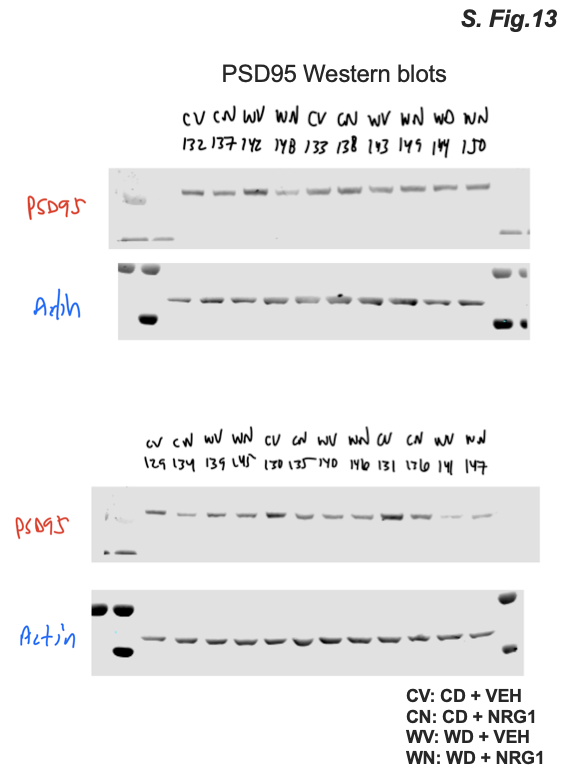

References

1 Dember WN, Fowler H. Spontaneous alternation after free and forced trials. *Can J Psychology Revue Can De Psychologie* 1959; 13: 151–154.

2 Deacon RMJ, Rawlins JNP. T-maze alternation in the rodent. *Nat Protoc* 2006; 1: 7–12.

3 Vega-Torres JD, Azadian M, Rios-Orsini RA, Reyes-Rivera AL, Ontiveros-Angel P, Figueroa JD. Adolescent Vulnerability to Heightened Emotional Reactivity and Anxiety After Brief Exposure to an Obesogenic Diet. *Front Neurosci-switz* 2020; 14: 562.
